# Regulation of endothelial intracellular adenosine via adenosine kinase epigenetically modulates vascular inflammation

**DOI:** 10.1038/s41467-017-00986-7

**Published:** 2017-10-16

**Authors:** Yiming Xu, Yong Wang, Siyuan Yan, Qiuhua Yang, Yaqi Zhou, Xianqiu Zeng, Zhiping Liu, Xiaofei An, Haroldo A. Toque, Zheng Dong, Xuejun Jiang, David J. Fulton, Neal L. Weintraub, Qinkai Li, Zsolt Bagi, Mei Hong, Detlev Boison, Chaodong Wu, Yuqing Huo

**Affiliations:** 1 Vascular Biology Center, Department of Cellular Biology and Anatomy, Medical College of Georgia, Augusta University, Augusta, GA 30912 USA; 2 School of Basic Medical Sciences, Guangzhou Medical University, Guangzhou, 511436 China; 3 College of Basic Medicine, Chengdu University of Traditional Chinese Medicine, Chengdu, 610075 China; 4 State Key Laboratory of Mycology, Institute of Microbiology, Chinese Academy of Science, Beijing, 100101 China; 5 Drug Discovery Center, Key Laboratory of Chemical Genomics, Peking University Shenzhen Graduate School, Shenzhen, 518055 China; 6 Department of Endocrinology, Jiangsu Province Hospital of Chinese Medicine, Nanjing, 210029 China; 7 Department of Pharmacology and Toxicology, Augusta University, Augusta, GA 30912 USA; 8 Department of Cellular Biology and Anatomy, Medical College of Georgia, Augusta University, Augusta, GA 30912 USA; 9 Robert S. Dow Neurobiology Laboratories, Legacy Research Institute, Portland, OR 97232 USA; 10 Department of Nutrition and Food Science, Texas A&M University, College Station, TX 77840 USA

## Abstract

The molecular mechanisms underlying vascular inflammation and associated inflammatory vascular diseases are not well defined. Here we show that endothelial intracellular adenosine and its key regulator adenosine kinase (ADK) play important roles in vascular inflammation. Pro-inflammatory stimuli lead to endothelial inflammation by increasing endothelial ADK expression, reducing the level of intracellular adenosine in endothelial cells, and activating the transmethylation pathway through increasing the association of ADK with S-adenosylhomocysteine (SAH) hydrolase (SAHH). Increasing intracellular adenosine by genetic ADK knockdown or exogenous adenosine reduces activation of the transmethylation pathway and attenuates the endothelial inflammatory response. In addition, loss of endothelial ADK in mice leads to reduced atherosclerosis and affords protection against ischemia/reperfusion injury of the cerebral cortex. Taken together, these results demonstrate that intracellular adenosine, which is controlled by the key molecular regulator ADK, influences endothelial inflammation and vascular inflammatory diseases.

## Abstract (web summary)

The molecular mechanisms underlying vascular inflammation are unclear. Here the authors show that pro-inflammatory stimuli lead to endothelial inflammation by increasing adenosine kinase expression, and that its knockdown in endothelial cells inhibits atherosclerosis and cerebral ischemic injury in mice.

## Introduction

Adenosine is an endogenous purine ribonucleoside that is generated and released by many cell types during inflammation. Increased levels of adenosine can reduce leukocyte activation and recruitment by acting on an array of adenosine receptors^1–3^. However, the role of adenosine in endothelial inflammation and its molecular mechanisms have remained poorly defined. Furthermore, it is urgent to pursue new pharmacological approaches for elevating endogenous adenosine to treat inflammatory vascular diseases.

Adenosine is produced both inside and outside the cell through the breakdown of adenosine triphosphate (ATP)^4^. In addition to ATP degradation, intracellular adenosine can also be formed via the transmethylation pathway^4^. The S-adenosylmethionine (SAM)-dependent transmethylation reaction generates S-adenosylhomocysteine (SAH), which is a potent feed-back inhibitor of methyltransferases. SAH is subsequently hydrolyzed by SAH hydrolase (SAHH) to adenosine and L-homocysteine (Hcy) (Supplementary Fig. 1a). This reaction is driven by low levels of adenosine, whereas increased levels of adenosine shift the equilibrium toward increased formation of SAH and thereby constrain transmethylation reactions^4^. Inhibition of the transmethylation pathway by SAHH inhibitors has been shown to promote immunosuppression in T cells and macrophages^5, 6^. Whether intracellular adenosine can regulate endothelial inflammation by modulating pathways of transmethylation is not yet known and is the goal of the current study.

The level of intracellular adenosine is regulated by the metabolism of adenosine to 5′-adenosine monophosphate (AMP) via adenosine kinase (ADK) or to inosine via adenosine deaminase (ADA). As a low capacity, high affinity enzyme with a much lower Michaelis constant (1 mM), ADK is regarded as the principal enzyme in regulating intracellular adenosine concentrations under physiological conditions^4^. Pharmacologically, ADK inhibitors have been shown to raise adenosine levels, resulting in beneficial responses including neuroprotection, seizure suppression, and anti-psychotic effects^4, 7, 8^. Herein, we investigated the impact of targeting endothelial ADK in order to determine the importance of intracellular adenosine in endothelial inflammation. Our study showed that ADK inactivation elevates intracellular adenosine and inhibits inflammatory response via decreasing the pro-inflammatory stimuli-induced hypermethylation of histone H3 at lysine 4 (H3K4), indicating the therapeutic potential of targeting ADK for inflammatory vascular disorders.

## Results

### Loss of endothelial ADK suppresses endothelial inflammation

**Fig. 1:**
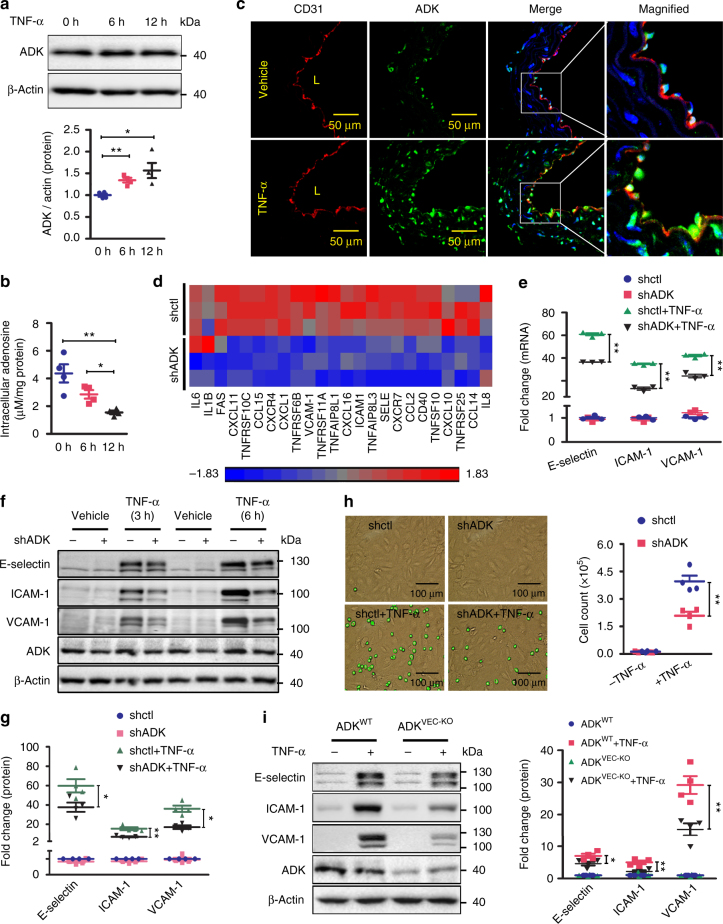
ADK deficiency suppresses endothelial inflammation in vitro. **a** Western blot detection of ADK in HUVECs treated with TNF-α at 10 ng/ml (*n* = 4). **b** Quantification of intracellular adenosine by HPLC in HUVECs exposed to TNF-α at 10 ng/ml (*n* = 4). **c** Representative images of ADK immunofluorescent (IF, *green*) staining on aortic endothelium (areas indicated with CD31 staining, *red*) of WT mice treated with vehicle or TNF-α (10 ng per gram mouse body weight for 5 h). *L* indicates luminal area (*scale bar*, 50 µm; *n* = 5–6 mice per group). **d** Heat map of inflammation-related genes in ADK KD or Ctrl HUVECs (*n* = 3). **e** RT-PCR analysis of mRNA levels of adhesion molecules in TNF-α (10 ng/ml for 2 h)-treated ADK KD or Ctrl HUVECs (*n* = 3). **f** Western blot detection of adhesion molecules in ADK KD or Ctrl HUVECs treated with TNF-α at 10 ng/ml for 3 or 6 h. Images are representative of four independent experiments. **g** Quantification of adhesion molecular expression in ADK KD or Ctrl HUVECs treated with TNF-α at 10 ng/ml for 6 h (*n* = 4). **h** Representative images and quantification of monocyte adhesion on TNF-α (10 ng/ml for 4 h)-treated ADK KD or Ctrl HUVECs (*scale bar*, 100 µm; *n* = 4). **i** Western blot detection and densitometric quantification of adhesion molecule expression in 10 ng/ml TNF-α-treated MAECs isolated from ADK^WT^ or ADK^VEC-KO^ mice (*n* = 4). For all *bar graphs*, data are the mean ± SEM, *P < 0.05 and **P < 0.01 (one-way ANOVA with Tukey’s post hoc test for **a**, **b**; unpaired, two-tailed Student’s *t*-test for **e**, **i**)

Tumor necrosis factor (TNF)-α is a vital mediator of systemic inflammation and immune responses. One of the major cellular targets for TNF-α inflammatory action is the vascular endothelium, where TNF-α exhibits inflammatory responses by expressing adhesion molecules and secreting pro-inflammatory cytokines. We exposed confluent human umbilical vein endothelial cells (HUVECs) to 10 ng/ml TNF-α for 0–12 h. We found that TNF-α treatment did not affect the mRNA level but upregulated the protein level of ADK (Fig. 1a). Consistent with the enhanced level of adenosine-metabolizing enzyme, intracellular adenosine levels significantly decreased in HUVECs exposed to TNF-α for 6 and 12 h (Fig. 1b). In addition, immunostaining showed an increase of ADK in aortic endothelium of TNF-α-treated mice when compared with that of vehicle-treated mice (Fig. 1c and Supplementary Fig. 2a). To investigate the role of intracellular adenosine in endothelial inflammation, ADK was silenced in HUVECs using an adenovirus-delivered short hairpin RNA (shRNA) approach that significantly reduced both mRNA and protein levels of ADK (Supplementary Fig. 2b, c), and resulted in elevated intracellular adenosine (Supplementary Fig. 2d). To examine the impact of reduced ADK expression on the pattern of endothelial gene expression, we performed microarrays using mRNA extracted from HUVECs transduced with ADK knockdown (KD) and control (Ctrl) adenoviruses. Using gene set enrichment analysis we analyzed the expression of genes linked to the endothelial cell inflammatory response (Fig. 1d). The expression of 24 inflammatory genes, including E-selectin, ICAM-1, and VCAM-1, were significantly lower in ADK KD HUVECs than in Ctrl HUVECs. Furthermore, ADK KD blocked TNF-α- and IL-1β-induced E-selectin, ICAM-1, and VCAM-1 expression at the mRNA and protein levels (Fig. 1e–g and Supplementary Fig. 2e). The number of monocytes adhering to TNF-α-stimulated ADK-KD HUVECs was reduced by 47% compared with that of Ctrl HUVECs (Fig. 1h). Two selective ADK inhibitors, 5′-iodotubercidin (ITU) and ABT-702, at doses of 10 and 2 µM, respectively, inhibited TNF-α-induced expression of E-selectin, ICAM-1, and VCAM-1 (Supplementary Fig. 2f, g). In addition, ADK KD blocked TNF-α-stimulated mRNA expression of IL-6, MCP-1, and IL-8 (Supplementary Fig. 2h). To investigate the anti-inflammatory effects of intracellular adenosine in primary endothelial cells ex vivo, endothelial cell-specific ADK knockout (ADK^VEC-KO^) and control (ADK^WT^) mice were generated, and mouse aortic endothelial cells (MAECs) were isolated (Supplementary Fig. 1b). Endothelial ADK deficiency significantly decreased ADK level (Supplementary Fig. 1c) and accordingly increased the intracellular adenosine level (Supplementary Fig. 1d) but had little effect on the systemic adenosine level (Supplementary Fig. 1e–h). Consistent with the in vitro data from HUVECs, ADK deficiency also dampened the TNF-α-induced expression of adhesion molecules in MAECs (Fig. 1i).

Since intracellular and extracellular pools of adenosine are exchanged dynamically through the equilibrative nucleoside transporters (ENTs)^9^, we next tested whether adenosine receptors are responsible for the suppression of adhesion molecule expression upon ADK KD. Among the four adenosine receptors, A_2A_Rs and A_2B_Rs are prominently expressed in endothelial cells, and their expression levels were significantly upregulated by ADK KD^10^. We treated ADK-KD and -Ctrl HUVECs with vehicle or a combination of A_2A_R (ZM241385) and A_2B_R (MRS1754) antagonists, followed by incubation with TNF-α for 4 h. The combination of these two antagonists at concentrations that effectively lowered intracellular cAMP levels^10^ did not affect TNF-α-induced expression of E-selectin, ICAM-1, or VCAM-1 in ADK-KD or -Ctrl HUVECs (Supplementary Fig. 3a). Moreover, using a siRNA interference strategy (Supplementary Fig. 2i), we observed that the anti-inflammatory effects of ADK KD were still preserved in A_2A_R or A_2B_R KD endothelial cells (Supplementary Fig. 3b, c). Altogether, these findings suggest that the anti-inflammatory effects of elevated intracellular adenosine by ADK KD are at least partially adenosine receptor-independent in endothelial cells.

Adenosine can also be converted inside the cell to inosine by ADA^11^. Therefore, we investigated whether the purine intermediate inosine inhibits TNF-α-induced production of E-selectin, ICAM-1, or VCAM-1. Treatment with inosine in concentrations ranging from 0.1–1 mM did not inhibit TNF-α-induced expression of adhesion molecules (Supplementary Fig. 3d), suggesting that the anti-inflammatory effects of intracellular adenosine are independent of its conversion to inosine.

### Intracellular adenosine suppresses endothelial inflammation

**Fig. 2:**
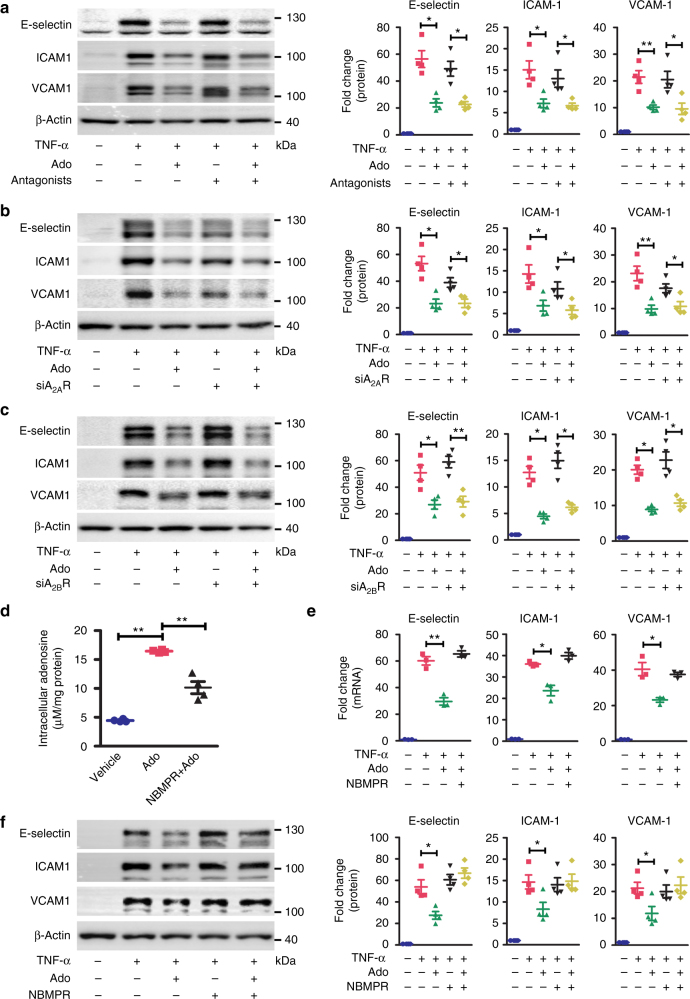
Adenosine-induced suppression of endothelial inflammation requires adenosine uptake. **a** Western blot detection and densitometric quantification of adhesion molecule expression in HUVECs. HUVECs, pretreated for 30 min with both 5 µM ZM 241385 and 5 µM MRS 1754 were incubated with 100 µM adenosine for 30 min and then stimulated with TNF-α at 10 ng/ml for 4 h (*n* = 4). **b** Western blot detection and densitometric quantification of adhesion molecule expression in HUVECs. HUVECs, transiently transfected with control or A_2A_R siRNA, were pretreated for 30 min with 100 µM adenosine and then stimulated with 10 ng/ml TNF-α for 4 h (*n* = 4). **c** Western blot detection and densitometric quantification of adhesion molecule expression in HUVECs. HUVECs, transiently transfected with control or A_2B_R siRNA, were pretreated for 30 min with 100 µM adenosine and then stimulated with 10 ng/ml TNF-α for 4 h (*n* = 4). **d** Quantification of intracellular adenosine by HPLC in adenosine (100 µM for 1 h)-treated HUVECs and in adenosine (100 µM for 1 h)-treated HUVECs preincubated with NBMPR at 10 µM for 30 min (*n* = 4). **e** Real-time-PCR (RT-PCR) analysis of mRNA levels of adhesion molecules in HUVECs. HUVECs, pretreated for 30 min with 10 µM NBMPR, were incubated with 100 µM adenosine for 30 min and then stimulated with TNF-α at 10 ng/ml for 2 h (*n* = 3). **f** Western blot detection and densitometric quantification of adhesion molecule expression in HUVECs. HUVECs, pretreated for 30 min with 10 µM NBMPR, were incubated with 100 µM adenosine for 30 min and then stimulated with TNF-α at 10 ng/ml for 4 h (*n* = 4). All images are representative. For all *bar graphs*, data are the mean ± SEM, *P < 0.05 and **P < 0.01 (one-way ANOVA with Tukey’s post hoc test)

Having demonstrated that elevated level of intracellular adenosine inhibits endothelial inflammation, we further studied whether this intracellular mechanism is also applied to the anti-inflammatory effect of exogenous adenosine. HUVECs were treated with exogenous adenosine at 20–100 µM, a concentration range observed in ischemic or inflamed tissues^12^. Exogenous adenosine dose-dependently decreased TNF-α-induced expression of E-selectin, ICAM-1, and VCAM-1 (Supplementary Fig. 4a) while inhibiting TNF-α-induced THP-1 cell adhesion to HUVECs (Supplementary Fig. 4b). The combination of the A_2A_R antagonist ZM241385 and the A_2B_R antagonist MRS1754 did not block the effect of adenosine on suppressing the TNFα-induced expression of E-selectin, ICAM-1, and VCAM-1 in HUVECs (Fig. 2a). Furthermore, neither A_2A_R nor A_2B_R KD compromised the ability of adenosine to suppress adhesion molecule expression (Fig. 2b, c). Altogether, these results suggest that mechanisms other than adenosine receptor activation mediate the anti-inflammatory effect of exogenous adenosine in endothelial cells.

To test whether intracellular translocation of adenosine is required for the anti-inflammatory effects of exogenous adenosine, we pre-incubated HUVECs with the ENT inhibitors, nitrobenzylthioinosine (NBMPR) and dipyridamole, to block the transmembrane transport of adenosine (Fig. 2d–f and Supplementary Fig. 4c). Exogenous adenosine dramatically increased the levels of intracellular adenosine in HUVECs (Fig. 2d) and decreased the mRNA (Fig. 2e) and protein (Fig. 2f) expression of E-selectin, ICAM-1, and VCAM-1. Pretreatment with NBMPR, a specific ENT1 inhibitor, prior to the addition of adenosine not only reversed adenosine-augmented intracellular adenosine concentration, but also reversed the inhibitory effect of adenosine on E-selectin, ICAM-1, and VCAM-1 expression (Fig. 2d–f). Moreover, the nonselective ENT inhibitor dipyridamole also counteracted the inhibitory effect of adenosine on ICAM-1 and VCAM-1 expression (Supplementary Fig. 4c). Altogether, these results suggest that the intracellular translocation of adenosine is required for extracellular adenosine to inhibit TNF-α-induced adhesion molecule expression in HUVECs.

### ADK deficiency suppresses endothelial inflammation in vivo

**Fig. 3:**
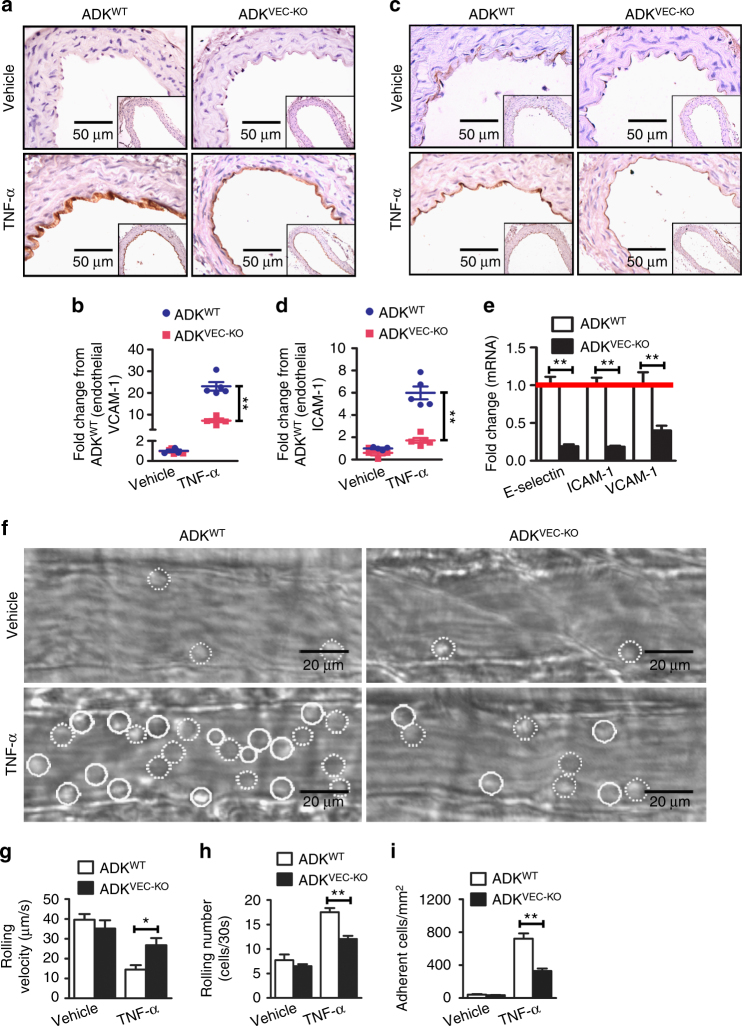
ADK deficiency suppresses endothelial inflammation in vivo. **a** Representative images for immunohistochemical staining of VCAM-1 in aortas from TNF-α-treated ADK^WT^ and ADK^VEC-KO^ mice (*scale bar*, 50 µm). **b** Quantification of VCAM-1 expression in aortas from TNF-α-treated ADK^WT^ and ADK^VEC-KO^ mice and the graph shows the data as fold change compared with the vehicle-treated ADK^WT^ mice (*n* = 4–5 mice per group). **c** Representative images for immunohistochemical staining of ICAM-1 in aortas from TNF-α-treated ADK^WT^ and ADK^VEC-KO^ mice (*scale bar*, 50 µm). **d** Quantification of ICAM-1 expression in aortas from TNF-α-treated ADK^WT^ and ADK^VEC-KO^ mice and the graph shows the data as fold change compared with the vehicle-treated ADK^WT^ mice (*n* = 4–5 mice per group). **e** RT-PCR analysis of mRNA levels of adhesion molecules in aortas from TNF-α-treated ADK^WT^ and ADK^VEC-KO^ mice (*n* = 5 mice per group). **f** Representative images of leukocyte rolling and adhesion on the endothelium of postcapillary venules in the cremaster muscles of vehicle or TNF-α-treated ADK^WT^ and ADK^VEC-KO^ mice. Rolling and adherent cells are indicated with *dotted* and *solid circle line*, respectively (*scale bar*, 20 µm). **g** Rolling velocity of leukocytes (at least 60 rolling leukocytes in 12 postcapillary venules of four mice per group). **h** Rolling number of leukocytes (at least 24 postcapillary venule segments in four mice per group). **i** Number of adherent leukocytes (at least 24 postcapillary venule segments in four mice per group). For *bar graphs*, data are the mean ± SEM, *P < 0.05 and **P < 0.01 (unpaired, two-tailed Student’s *t*-test)

To investigate the anti-inflammatory effects of intracellular adenosine in endothelial cells in vivo, ADK^VEC-KO^ and control ADK^WT^ mice were injected with TNF-α (10 µg/ml) intraperitoneally to induce vascular inflammation in vivo. Five hours after TNF-α treatment, the expression of ICAM-1 and VCAM-1 in aortic endothelial cells was elevated in ADK^WT^ mice, but markedly blunted in ADK^VEC-KO^ mice (Fig. 3a–e).

To determine the functional consequences of endothelial ADK deficiency in vivo, we evaluated leukocyte rolling and adhesion in the endothelium of postcapillary venules of mouse cremaster muscle using intravital microscopy. Mild trauma caused by the exteriorization of the cremaster muscle leads to fast leukocyte rolling. In ADK^VEC-KO^ mice, the number of fast rolling leukocytes and rolling velocity were comparable to that in ADK^WT^ mice (Fig. 3f–h). TNF-α treatment stimulated the expression of adhesion molecules on the endothelium and induced slow rolling and adhesion of leukocytes in ADK^WT^ mice, which was reduced by 40–50% in ADK^VEC-KO^ mice (Fig. 3f–i and Supplementary Movies 1–4), indicating diminished activation of the endothelium in ADK^VEC-KO^ mice.

### Intracellular adenosine inhibits the methylation of H3K4

**Fig. 4:**
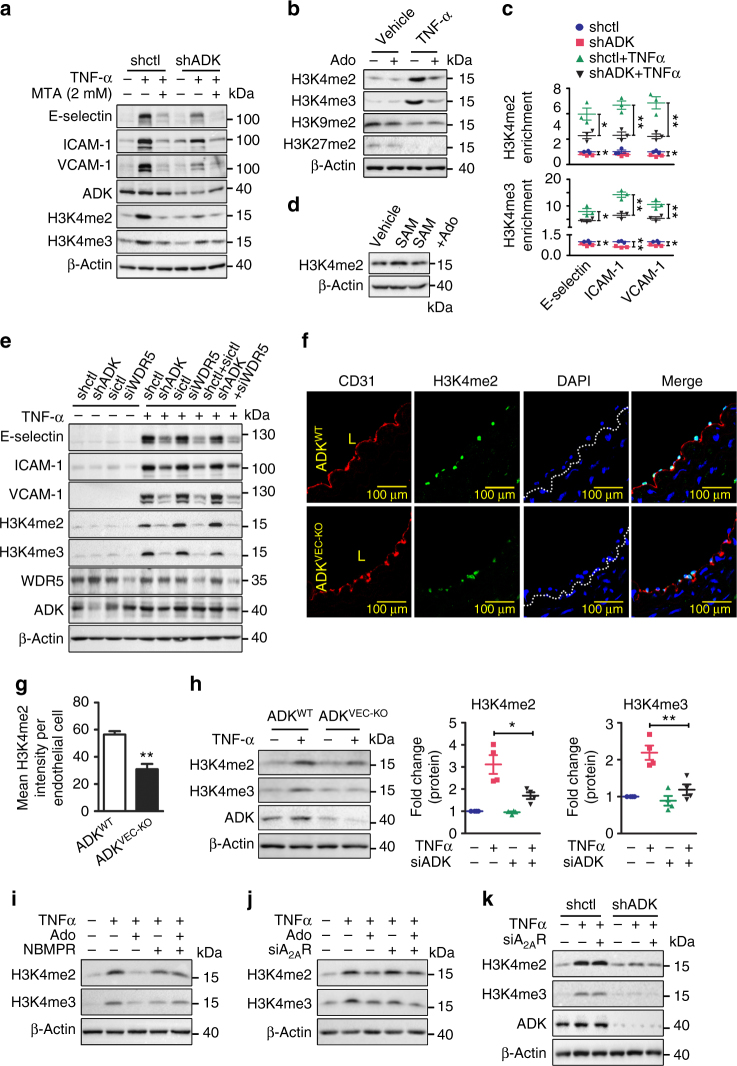
Intracellular adenosine decreases the methylation level of H3K4. **a** Western blot detection of adhesion molecules and H3K4 methylation in TNF-α (10 ng/ml for 4 h)-treated ADK KD or Ctrl HUVECs pretreated with 2 mM MTA for 30 min (*n* = 4). **b** Western blot detection of methylation levels of H3K4, H3K9, and H3K27 in TNF-α (10 ng/ml for 12 h)-treated HUVECs pretreated with 100 µM adenosine for 30 min (*n* = 5). **c** ChIP assay showing H3K4 methylation on promoters of adhesion molecules in TNF-α (10 ng/ml for 4 h)-treated ADK KD or Ctrl HUVECs (*n* = 3). **d** Western blot detection of H3K4me2 in HUVEC whole-protein lysate supplemented with 1 mg/ml SAM or SAM together with 10 µM adenosine for 60 min (*n* = 4). **e** Western blot detection of adhesion molecules and H3K4 methylation in TNF-α (10 ng/ml for 4 h)-treated ADK KD or Ctrl HUVECs transiently transfected with control or WDR5 siRNA (*n* = 3). **f** Immunofluorescent (*IF*) staining of H3K4me2 on aortic endothelium (areas indicated with CD31 staining, *red*) from TNF-α-treated ADK^WT^ and ADK^VEC-KO^ mice. *L* indicates luminal area of aorta (*scale bar*, 100 µm). **g** Quantification of H3K4me2 on aortic endothelium from TNF-α-treated ADK^WT^ and ADK^VEC-KO^ mice (*n* = 5 mice per group). **h** Western blot detection and densitometric quantification of H3K4 methylation in TNF-α (10 ng/ml for 4 h)-treated MAECs isolated from ADK^WT^ and ADK^VEC-KO^ mice (*n* = 4). **i** Western blot detection of H3K4 methylation in HUVECs. HUVECs, pretreated for 30 min with 10 µM NBMPR, were incubated with 100 µM adenosine for 30 min and then stimulated with TNF-α at 10 ng/ml for 4 h (*n* = 3). **j** Western blot detection of H3K4 methylation in TNF-α (10 ng/ml for 4 h)-treated A_2A_R KD or Ctrl HUVECs pretreated with 100 µM adenosine for 30 min (*n* = 4). **k** Western blot detection of H3K4 methylation in TNF-α (10 ng/ml for 4 h)-treated ADK KD or Ctrl HUVECs transiently transfected with control or A_2A_R siRNA (*n* = 4). For all *bar graphs*, data are the mean ± SEM, *P < 0.05 and **P < 0.01 (unpaired, two-tailed Student’s *t*-test)

Increased intracellular adenosine suppresses SAM-dependent methyltransferases, thereby exerting epigenetic effects through interference with the transmethylation pathway^4, 13^. Methylation of histone H3 at lysine 4 (H3K4) is a transcriptional activating mark that is associated with increased gene expression and vascular inflammation^14, 15^. TNF-α treatment for 6 h significantly enhanced the dimethylation (H3K4me2) and trimethylation (H3K4me3) of histone H3 at lysine 4 in HUVECs, which was attenuated by ADK KD (Fig. 4a and Supplementary Fig. 5a). To determine the functional importance of H3K4 methylation in endothelial inflammation, HUVECs were treated with 2 mM 5′-deoxy-5′ (methylthio) adenosine (MTA), a non-selective histone methylation inhibitor. Pretreatment with MTA at 2 mM diminished both histone methylation and TNF-α-induced expression of E-selectin, ICAM-1, and VCAM-1 (Fig. 4a and Supplementary Fig. 5a). In addition, pretreatment with adenosine before addition of TNF-α decreased the levels of H3K4me2 and H3K4me3 while not affecting the dimethylation of histone H3 at lysine 9 (H3K9me2) or lysine 27 (H3K27me2) (Fig. 4b and Supplementary Fig. 5b); dimethylation of histone H3 at lysine 36 (H3K36me2) was undetectable. To determine how these changes impact the levels of methylated histone 3 at the promoters of inflammatory genes, we next performed chromatin immunoprecipitation (ChIP) assays. We found that TNF-α increased the binding of H3K4me2 and H3K4me3 to the E-selectin, ICAM-1, and VCAM-1 promoters (Fig. 4c), whereas the levels of basal and TNF-α-induced H3K4me2 and H3K4me3 at these promoters were diminished in ADK-KD HUVECs compared to ADK-Ctrl HUVECs (Fig. 4c).

To investigate whether adenosine directly affects H3K4 methylation, HUVEC whole-cell lysates were extracted and incubated with SAM together with adenosine or vehicle. SAM treatment markedly increased the level of H3K4me2, which was blocked by the addition of adenosine (Fig. 4d and Supplementary Fig. 5c). Knockdown of WDR5 (a core subunit of histone H3K4 methyltransferase^16^) blocked H3K4 transmethylation and mimicked the anti-inflammatory effects of ADK KD in endothelial cells (Fig. 4e). Additionally, simultaneous knockdown of both WDR5 and ADK achieved an effect similar to knockdown of either molecule, suggesting common mechanisms of action. Moreover, in ADK^VEC-KO^ mice, TNF-α injection produced lower levels of endothelial H3K4me2 compared with ADK^WT^ mice (Fig. 4f, g). In MAECs isolated from ADK^VEC-KO^ mice, the ability of TNF-α to induce di- and tri-methylation of H3K4 was significantly impaired (Fig. 4h). Altogether, these data suggest that the anti-inflammatory effects of intracellular adenosine in endothelial cells are mediated through inhibition of H3K4 methylation.

To further investigate the mechanisms of adenosine-induced inhibition of H3K4 methylation, NBMPR was employed to block the transport of adenosine into cells. In the presence of NBMPR, adenosine failed to inhibit H3K4me2 and H3K4me3 (Fig. 4i and Supplementary Fig. 5d). In contrast, A_2A_R KD (Fig. 4j and Supplementary Fig. 5e), A_2B_R KD (Supplementary Fig. 5a), or the combination of ZM241385 and MRS1754 (Supplementary Fig. 5b) had no effect on the levels of H3K4me2 and H3K4me3 in either vehicle- or adenosine-treated HUVECs. Similar results were observed in ADK KD cells (Fig. 4k and Supplementary Figs 5f, 6c, d). Altogether, these data suggest that adenosine blocks H3K4 methylation via an intracellular mechanism.

### ADK binds with SAHH to facilitate the methylation of H3K4

**Fig. 5:**
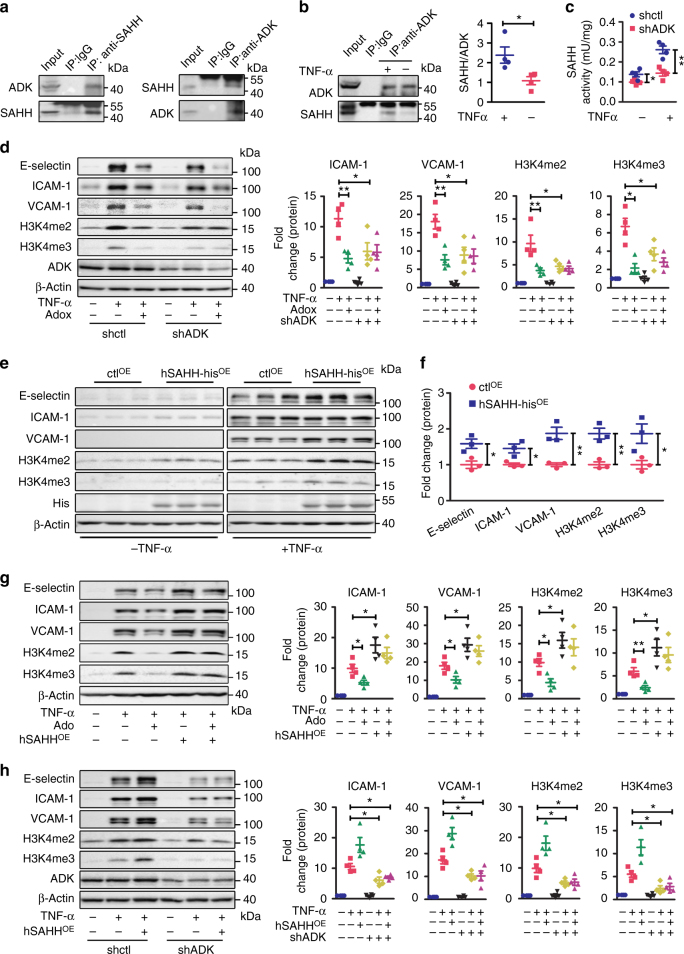
ADK binds with SAHH to facilitate H3K4 methylation and endothelial inflammation. **a** Co-immunoprecipitation (*co-IP*) of overexpressed SAHH and endogenous ADK in HUVECs. *Upper*: IP was performed with mouse anti-His antibody followed by immunoblotting with rabbit anti-ADK antibody and rabbit anti-SAHH antibody. *Lower*: IP was performed with goat anti-ADK antibody followed by immunoblotting with rabbit anti-SAHH antibody and rabbit anti-ADK antibody (*n* = 4). **b** Co-IP of endogenous SAHH and ADK in vehicle or TNF-α (10 ng/ml for 12 h)-treated HUVECs. *Upper*: IP was performed with goat anti-ADK antibody followed by immunoblotting with rabbit anti-SAHH antibody and rabbit anti-ADK antibody. *Lower*, Quantification of ADK–SAHH complex amount; control indicates no TNF-α treatment (*n* = 4). **c** The activity of SAHH in TNF-α (10 ng/ml for 12 h)-treated ADK KD or Ctrl HUVECs (*n* = 4). **d** Western blot detection and densitometric quantification of adhesion molecule expression and H3K4 methylation in TNF-α (10 ng/ml for 4 h)-treated ADK KD or Ctrl HUVECs preincubated with 20 µM adenosine-2′, 3′-dialdehyde (Adox) for 30 min (*n* = 4). **e** Western blot detection of adhesion molecules and H3K4 methylation in vehicle or TNF-α (10 ng/ml for 4 h)-treated Ctrl- or SAHH-overexpressing HUVECs (*n* = 3). **f** Densitometric quantification of adhesion molecule expression and H3K4 methylation in TNF-α-treated Ctrl- or SAHH-overexpressing HUVECs (*n* = 3). **g** Western blot detection and densitometric quantification of adhesion molecule expression and H3K4 methylation in TNF-α (10 ng/ml for 4 h)-treated vehicle or adenosine pre-incubated control or SAHH-overexpressing HUVECs (*n* = 4). **h** Western blot detection and densitometric quantification of adhesion molecule expression and H3K4 methylation in TNF-α (10 ng/ml for 4 h)-treated Ctrl or ADK KD/SAHH-overexpressing HUVECs (*n* = 4). All images are representative. For all *bar graphs*, data are the mean ± SEM, *P < 0.05 and **P < 0.01 (Unpaired, two-tailed Student’s *t*-test for **b**, **c**; one-way ANOVA with Tukey’s post hoc test for **d**, and **f**–**h**)

In plants, ADK has been shown to bind SAHH, which enables the rapid degradation of the adenosine produced by SAHH^17^. To investigate whether ADK and SAHH might interact in endothelial cells, co-immunoprecipitation (co-IP) assays were performed using protein extracts from HUVECs overexpressing His-tagged SAHH with an anti-His antibody or an anti-ADK antibody (Fig. 5a). ADK was present in complexes precipitated by the anti-His antibody and in reciprocal experiments; SAHH was present in immune complexes concentrated by the anti-ADK antibody (Fig. 5a). In non-transfected cells, an interaction between endogenous ADK and SAHH was detected, and this association was further strengthened by TNF-α treatment (Fig. 5b). The ADK KD HUVECs had significantly decreased SAHH activity in the absence or presence of TNF-α (Fig. 5c), without affecting SAHH protein levels (Supplementary Fig. 6e), suggesting that the interaction of SAHH and ADK has functional consequences. We, therefore, investigated whether SAHH regulates adenosine-induced inhibition of H3K4 methylation and adhesion molecule expression in endothelial cells. Treatment with the SAHH inhibitor, Adenosine-2′,3′-dialdehyde (Adox), inhibited TNF-α-induced expression of E-selectin, ICAM-1, and VCAM-1(Fig. 5d) and blocked the effects of exogenous adenosine on these adhesion molecules (Supplementary Fig. 7a). Adox also reduced TNF-α-induced di- and tri-methylation of H3K4 and blocked the effects of exogenous adenosine and ADK KD on H3K4 methylation (Fig. 5d and Supplementary Fig. 7a). Conversely, overexpression of SAHH upregulated the expression of E-selectin, ICAM-1, and VCAM-1, and increased the levels of H3K4me2 and H3K4me3 in the absence or presence of TNF-α (Fig. 5e, f). Furthermore, SAHH overexpression negated the inhibitory effects of adenosine on TNF-α-induced expression of E-selectin, ICAM-1, and VCAM-1 as well as H3K4 methylation (Fig. 5g). However, SAHH overexpression did not affect the ability of ADK KD to suppress TNF-α-mediated increases in adhesion molecule expression and H3K4 methylation (Fig. 5h), indicating that the ADK-mediated clearance of intracellular adenosine is essential for SAHH to promote adhesion molecule expression and H3K4 methylation. As the substrate of the SAHH-mediated reaction, SAH is a strong inhibitor of SAM-dependent methyltransferase reactions^18^. Similar to adenosine treatment or ADK KD, SAH pretreatment significantly inhibited the ability of TNF-α to increase E-selectin, ICAM-1, and VCAM-1 levels (Supplementary Fig. 7b, c). Moreover, this effect correlates with reduced levels of H3K4me2 and H3K4me3 (Supplementary Fig. 7b, c). Collectively, these findings suggest that the anti-inflammatory effects of intracellular adenosine in endothelial cells is driven, at least in part, by the inhibition of SAHH-mediated hydrolysis of SAH, which then interferes with H3K4 methylation.

### Deficiency of endothelial ADK reduces atherogenesis

**Fig. 6:**
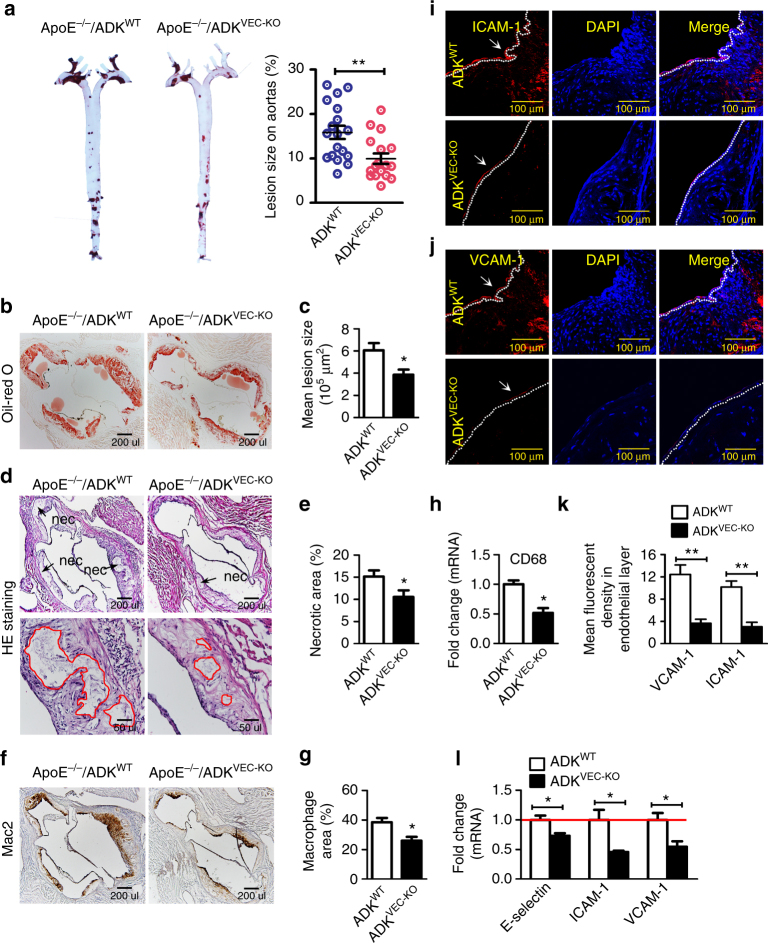
Endothelial-specific ADK deficiency prevents atherogenesis. **a** Oil Red O-stained en face aortic preparations from ApoE^−/−^/ADK^WT^ and ApoE^−/−^/ADK^VEC-KO^ mice and quantification of the Oil Red O-stained areas (*n* = 16–18 mice per group). **b**, **c** Oil Red O staining of aortic roots and quantification of lesion area in aortic roots of ApoE^−/−^/ADK^WT^ and ApoE^−/−^/ADK^VEC-KO^ mice (*scale bar*: 200 µm for **b**; *n* = 6 mice per group). **d**, **e** HE staining of aortic roots and quantification of necrotic areas in aortic roots of ApoE^−/−^/ADK^WT^ and ApoE^−/−^/ADK^VEC-KO^ mice (*scale bar*: 200 µm for *upper images* and 50 µm for *lower images*; *n* = 6 mice per group). *Red lines* show the boundary of necrotic cores. **f**–**h** IHC staining of Mac2 (**f**; *scale bar*: 200 µm), quantification of Mac2^+^ areas in aortic roots (**g**, *n* = 6 mice per group), and RT-PCR analysis of mRNA levels of CD68 (**h**, *n* = 5 per group) in aortic arches of ApoE^−/−^/ADK^WT^ and ApoE^−/−^/ADK^VEC-KO^ mice. **i**–**k** Immunofluorescent staining of ICAM-1 (**i**; *scale bar*: 100 µm) and VCAM-1 (**j**; *scale bar*: 100 µm) in aortic roots, and quantification of ICAM-1 and VCAM-1 expression in endothelial layer in aortic roots from ApoE^−/−^/ADK^WT^ and ApoE^−/−^/ADK^VEC-KO^ mice (**k**, *n* = 6 mice per group). **l** RT-PCR analysis of mRNA levels of adhesion molecules in aortic arches of ApoE^−/−^/ADK^WT^ and ApoE^−/−^/ADK^VEC-KO^ mice (*n* = 5 mice per group). All images are representative. For all *bar graphs*, data are the mean ± SEM, *P < 0.05 and **P < 0.01 (Unpaired, two-tailed Student’s *t*-test)

Recruitment of leukocytes into the arterial wall is one of the earliest events in atherosclerosis, and it is well established that immune cells contribute significantly to vascular inflammation and lesion development^19–22^. We, therefore, investigated whether selectively elevating intracellular adenosine in the endothelium via ADK knockout affects the development of atherogenesis in ApoE^−/−^ mice. Deletion of ADK in endothelial cells did not affect body weight or plasma cholesterol levels before (173.6 mg/dl vs. 154.8 mg/dl for ApoE^−/−^/ADK^WT^ and ApoE^−/−^/ADK^VEC-KO^) or after (808.1 mg/dl vs. 784.6 mg/dl for ApoE^−/−^/ADK^WT^ and ApoE^−/−^/ADK^VEC-KO^) introduction of a western diet. ApoE^−/−^/ADK^VEC-KO^ mice exhibited a significant reduction in atherosclerotic lesion size following 3 months of western diet (Fig. 6a–c). ADK deletion in endothelial cells also markedly decreased the necrotic area (Fig. 6d, e) and macrophage content in vascular lesions (Fig. 6f, g). This data is further supported by the reduced expression of mRNA encoding the macrophage marker CD68 in the aortic sinus of ApoE^−/−^/ADK^VEC-KO^ mice compared with ApoE^−/−^/ADK^WT^ mice (Fig. 6h). Immunohistochemistry staining (Fig. 6i–k) and real-time reverse transcriptase PCR (RT-PCR) assay (Fig. 6l) also showed a marked reduction in expression of ICAM-1 and VCAM-1 in the aortic sinus in ApoE^−/−^/ADK^VEC-KO^ mice compared with ApoE^−/−^/ADK^WT^ mice. Taken together, these data demonstrate that the endothelial ADK knockout confers athero-protection in ApoE^−/−^ mice in association with the downregulation of adhesion molecules.

### Endothelial ADK deficiency attenuates ischemic brain injury

**Fig. 7:**
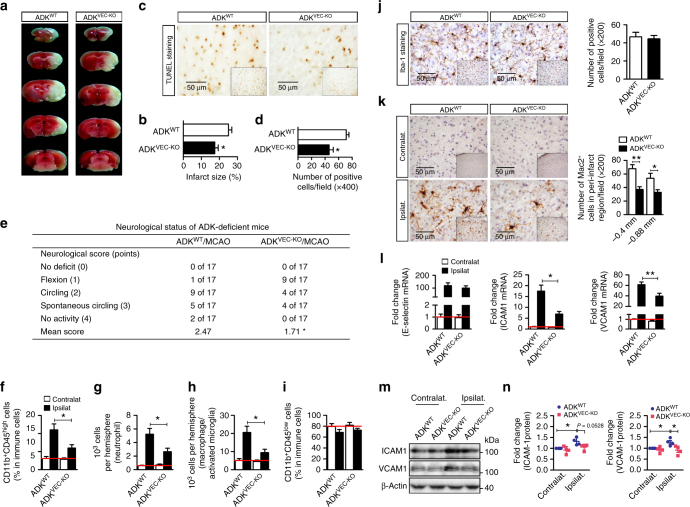
Endothelial ADK deficiency prevents cerebral ischemic injury. **a**, **b** 2,3,5-tripenyltetrazolium chloride (*TTC*) staining (**a**) and quantification of infarct size (**b**) in ADK^WT^ and ADK^VEC-KO^ mice (*n* = 10 mice per group). **c**, **d** Representative images of TUNEL staining (**c**; *scale bar*, 50 µm) and quantification of TUNEL-positive cells (**d**) in the penumbra of the ipsilateral ischemic cortex (on sections at a level 0.6 mm caudal to the bregma) of ADK^WT^ and ADK^VEC-KO^ mice (*n* = 5 per group). **e** Neurological status of ischemic ADK^WT^ and ADK^VEC-KO^ mice (*n* = 10 mice per group). **f**–**i** Flow cytometric analysis of inflammatory cell populations in the contralateral and ipsilateral brains of ADK^WT^ and ADK^VEC-KO^ mice 24 h post I/R (*n* = 6 mice per group). **f** The ratio of infiltrated leukocytes (CD11b^+^CD45^high^). **g** The number of infiltrated neutrophils (CD11b^+^CD45^high^Ly6G^+^). **h** The number of macrophages/activated microglia cells (CD11b^+^CD45^high^F4/80^+^). **i** The ratio of resident microglia cells (CD11b^+^CD45^low^). **j** IHC staining with Iba-1 antibody and quantification of Iba-1-positive resting microglia in contralateral brains of ADK^WT^ and ADK^VEC-KO^ mice 24 h post I/R. Representative images were taken in the contralateral cortex on sections at a level 0.5 mm caudal to Bregma (*scale bar*, 50 µm; *n* = 5 mice per group). **k** IHC staining and quantification of Mac2-positive macrophages/activated microglia cells on brain sections of ADK^WT^ and ADK^VEC-KO^ mice 24 h post I/R. Representative images were taken in the penumbra of the ipsilateral ischemic cortex and the corresponding contralateral cortex on sections at a level 0.4 mm caudal to the bregma (*scale bar*, 50 µm; *n* = 5 mice per group). **l** mRNA levels for adhesion molecules in brains of ADK^WT^ and ADK^VEC-KO^ mice 24 h post I/R (*n* = 6 mice per group). **m**, **n** Western blot detection (**m**) and densitometric quantification (**n**) of adhesion molecule expression in brains of ADK^WT^ and ADK^VEC-KO^ mice 24 h post I/R (*n* = 4 per group). For all *bar graphs*, data are the mean ± SEM, *P < 0.05 and **P < 0.01 (Unpaired, two-tailed Student’s *t*-test for **b**, **d**, and **g**–**m**; one-way ANOVA with Tukey’s post hoc test for **o**)

Endothelial adhesion molecules mediate leukocyte infiltration into the central nervous system during acute inflammation and contribute to neuron injury in ischemic stroke^23^. To investigate whether diminished adhesion molecule expression in the endothelial-specific ADK knockout mice confers neuroprotection in models of ischemic stroke, we performed a focal cerebral ischemia–reperfusion (I/R) operation in ADK^VEC-KO^ mice and ADK^WT^ littermates. Interestingly, 24 h after I/R, ADK^VEC-KO^ mice exhibited much smaller infarct size (Fig. 7a, b) and much fewer TUNEL-positive apoptotic cells (Fig. 7c, d) in the penumbra of the ipsilateral cortex than ADK^WT^ mice. Accordingly, ADK^VEC-KO^ mice showed improved neurological outcomes after I/R compared with control mice (Fig. 7e). To determine whether endothelial ADK mediates I/R-induced leukocyte brain infiltration to exacerbate an inflammatory response, immune cell populations in brain from ADK^VEC-KO^ and control mice were analyzed by flow cytometry (Supplementary Fig. 8). The recruitment of total leukocytes (CD11b^+^CD45^high^) and neutrophils (CD11b^+^CD45^high^Ly6G^+^) to the injured brain parenchyma was significantly less in ADK^VEC-KO^ mice 24 h post reperfusion (Fig. 7f–g). In addition, without affecting the ratio and number of resident microglia (CD11b^+^CD45^low^ cells in Fig. 7i and Iba-1^+^ cells in Fig. 7j), endothelial ADK deficiency dramatically decreased the number of macrophage/activated microglia (CD11b^+^CD45^high^F4/80^+^ cells in Fig. 7h and Mac2^+^ cells in Fig. 7k), indicating alleviated inflammatory response in ADK^VEC-KO^ mice. Furthermore, the upregulated mRNA and protein levels of ICAM-1 and VCAM-1 in forebrains by I/R were markedly attenuated in ADK^VEC-KO^ mice (Fig. 7l–n). Altogether, these data demonstrate that loss of endothelial ADK is sufficient to protect against I/R-induced cerebral injury that might result from the diminished endothelial inflammation and reduced leukocyte brain infiltration.

## Discussion

This study reveals a novel mechanism underlying the ability of adenosine to inhibit endothelial adhesion molecule expression and leukocyte—endothelial interactions, which is largely dependent upon the accumulation of intracellular adenosine. Increased intracellular adenosine represses flux through SAM-dependent transmethylation pathways, which leads to reduced H3K4 methylation and thus transcription and expression of genes encoding adhesion molecules (Supplementary Fig. 10). The translational significance of this pathway is shown in a novel mouse model where selective deletion of ADK in the endothelium confers protection against the development of atherosclerosis and cerebral ischemia/reperfusion injury. These findings provide new insights into a previously unrecognized ability of adenosine to alter epigenetic pathways regulating inflammation in endothelial cells and highlight the translational potential of targeting this pathway in the treatment of vascular disease.

Inflammatory stimuli have been shown to promote endothelial inflammation by altering the activity of transmethylation pathways. Transmethylation affects several cellular events, including macrophage and T-cell activation, and blockade of this pathway curtails inflammatory/autoimmune responses in immune cells^5, 6^. In the current study, we observed that TNF-α strengthened the association between ADK and SAHH and increased the activity of SAHH, suggesting that TNF-α potentiates the transmethylation pathway in endothelial cells. This concept is further strengthened by data showing that SAHH inhibition or SAHH overexpression can, respectively, suppress or enhance TNF-α stimulated inflammation in endothelium. A previous study from Barroso et al.^24, 25^ shows that SAHH inhibition or SAM treatment induced ICAM-1 and VCAM-1 expression. This apparent discrepancy may be related to different basal inflammatory status as Barroso et al. used resting rather than TNF-α-activated endothelial cells. For the genes undergoing transcriptional activation, their chromatins are actively remodeled into a more open conformation by post-translational changes on histone proteins that include a change in histone methylation. Methylation of histone 3 on lysine 4 (H3K4me2 and me3) or lysine 36 (H3K36me3) is associated with actively transcribed chromatin, whereas di- or tri-methylation of lysine 27 (H3K27) and/or lysine 9 (H3K9) generally correlates with repression^26^. Others have shown that the activation of NF-κB and the increased inflammatory response are associated with hypermethylation of H3K4 and hypomethylation of H3K9^14, 15^. In endothelial cells, we observed that TNF-α treatment promoted hypermethylation of H3K4 but hypomethylation of H3K9 and H3K27 (Fig. 4b and Supplementary Fig. 5b). In addition, depletion of WDR5, a core subunit of the histone methyltransferase complex responsible for H3K4 methylation^16^, hindered TNF-α-induced endothelial inflammation. Altogether, these data show that TNF-α promotes endothelial inflammation through potentiation of the transmethylation pathway and hypermethylation of H3K4. Although not examined in our study, it remains possible that the methylation of other non-histone proteins may also contribute to TNF-α-induced endothelial inflammation, including the lysine methylation of p65 which is important in the activation of the NF-κB pathway^27^.

Elevated intracellular adenosine is necessary to suppress endothelial inflammation. The ability of nucleoside transport inhibitors to negate the anti-inflammatory effects of exogenous adenosine in endothelial cells suggest that cytosolic translocation of adenosine is a requisite step. This concept is supported by the ability of ADK KD to increase the levels of intracellular adenosine, which also effectively represses endothelial inflammation. Higher levels of intracellular adenosine reduce the metabolism of SAH by SAHH, and the resulting increased levels of SAH, in turn, provide feedback inhibition of a wide range of methyltransferases^4^. In addition to this mechanism, the activity of SAHH may also be directly inhibited by adenosine^18^. Catalytic hydrolysis of SAH by SAHH requires NAD^+^ as a cofactor. Under in vitro conditions, adenosine can bind with high affinity to SAHH and reduce the binding of NAD^+^ and thus inactivate SAHH^18^. In endothelial cells, reduced expression of ADK or exogenous adenosine elicits the same effect as that of SAHH inhibition and WDR5 knockdown by inhibiting adhesion molecule expression and H3K4 methylation. These data suggest that intracellular adenosine promotes its anti-inflammatory effects via an epigenetic mechanism involving interruption of the transmethylation pathway and reduced H3K4 methylation.

Humans with natural deficiencies in the activity of ADK or SAHH exhibit similar clinical phenotypes^28, 29^, suggesting that ADK and SAHH regulate analogous physiological and/or developmental processes. In plant cells, ADK was observed to bind to SAHH^17^. We found that ADK also interacts with SAHH, suggesting a functional relationship between ADK and SAHH that has not been described before in human endothelial cells. The close association of these enzymes would enable the product of one enzyme to become the substrate of its neighboring enzyme with the net result of improving catalytic efficiency by limiting the local concentration of adenosine and thus negative regulation of SAHH and pathways of transmethylation.

Other possible mechanisms such as activation of adenosine receptors might also contribute to the suppressed endothelial inflammation by ADK deficiency. Although Bouma and colleagues previously could not delineate a clear role for adenosine receptors in restraining endothelial inflammation by exogenous adenosine^30^, many studies over the past several decades have demonstrated that adenosine counteracts the endothelial inflammatory response via A_2A_ and A_2B_ adenosine receptors. For example, Sands and colleagues have shown that upregulating A_2A_R gene expression in HUVECs suppressed inflammatory responses^31^. In a mouse arterial injury model, McPherson et al.^32^ demonstrated that an A_2A_R agonist inhibited adhesion molecule expression and arterial neointima formation. In vivo studies utilizing A_2B_R KO mice have shown that A_2B_R suppresses the expression of the adhesion molecules ICAM-1and E-selectin, which leads to decreased leukocyte rolling and adhesion^33^. Our findings that the elevated cAMP levels in ADK-deficient MAECs bring the possibility of autocrine activation of A_2A_R and/or A_2B_R upon ADK deficiency (Supplementary Fig. 9a). Additionally, the difference in TNF-α-induced adhesion molecule expression between ADK KD and control group in the absence of A_2A_R, although not significant, were slightly smaller than that in the presence of A_2A_R, indicating that autocrine activation of A_2A_R might slightly contribute to the anti-inflammatory effect of endothelial ADK KD. The almost completely abolished anti-inflammatory effect of exogenous adenosine by the ENT inhibitor, NBMPR (Fig. 2e, f), indicates that the intracellular mechanism is predominant in suppressing endothelial inflammation by adenosine. Apart from the autocrine effect, the paracrine activation of adenosine receptors might also be involved in the anti-inflammatory effect of endothelial ADK deletion in in vivo disease models. For examples, under in vivo conditions, adenosine that is released from endothelial cells in the setting of ADK deficiency may activate the A_2A_R or A_2B_R on adjacent cells such as leukocytes and macrophages to alleviate the vascular inflammation in atherosclerosis and ischemic stroke. For ischemic stroke, since activation of A_1_R on neurons is protective^34^, the adenosine released from ADK-deficient endothelial cells can also confer neuroprotection from stroke through an extracellular mechanism that involves activation of A_1_R on neurons.

A few studies have indicated that adenosine can promote apoptosis in certain types of cells^35^, which raises the possibility that the ability of adenosine to repress the expression of adhesion molecules is simply a consequence of apoptosis of those cells. However, HUVECs treated with 100 µM adenosine does not manifest apoptosis (Supplementary Fig. 9b, c), which is consistent with a previous report that incubation of HUVECs with adenosine up to 250 µM does not reduce cellular activity and integrity^30^. Additionally, inhibition of ADK in islet β-cells and cardiomyocytes promotes islet β-cell proliferation and cardiomyocyte growth^36, 37^. In line with these reports, we have observed that the accumulation of intracellular adenosine due to ADK KD did not cause apoptosis, but induced the proliferation of endothelial cells^10^. Thus, the anti-inflammatory effect of elevated intracellular adenosine is not a consequence of endothelial injury.

The results of our study suggest that impaired transmethylation contributes to the anti-inflammatory effect of intracellular adenosine in endothelial cells. However, suppression of transmethylation has also been reported to mediate endothelial dysfunction in response to elevated Hcy, which is another product of the SAHH reaction^38^. This apparent inconsistency is likely associated with the bidirectional fate of Hcy, which can either be fused with adenosine to form more SAH or recycled within the methionine cycle. Since SAHH facilitates the hydrolysis of SAH into Hcy and adenosine, the production of Hcy can be reduced upon SAHH inhibition. Nevertheless, the intracellular Hcy level in endothelial cells is not affected by TNF-α as well as exogenous adenosine or Adox-mediated SAHH inhibition (Supplementary Fig. 9d, e). These data further allude to the presence of the bidirectional fate of Hcy. Cacciapuoti et al.^39^ and Doshi et al.^40^ found that Hcy loading, although causing endothelial dysfunction, did not increased intracellular SAH and was not associated with a disruption in methylation status. Thus, it is likely that Hcy replenishes the methionine cycle, generates more SAM to accelerate transmethylation to promote inflammation and has negative effects on endothelial function.

We conclude that adenosine exerts its anti-inflammatory effects through epigenetic reprogramming of histone methylation, which limits the expression of adhesion molecules and leukocyte-endothelial interactions. Elevating intracellular adenosine selectively in endothelial cells by knocking out ADK had profound protective effects against atherosclerosis and I/R injury through effects on multiple cytokine and adhesion molecule pathways. The strategies to treat vascular inflammation by targeting a single cytokine or adhesion molecule (e.g., TNFα, IL-1, or ICAM-1) have met with limited success in humans, suggesting that a broader approach might be more effective. Therefore, ADK, which can impact multiple pathways via epigenetic changes, could be a promising target for treating vascular inflammatory diseases.

## Methods

### Mouse generation and breeding

The use of mice was in accordance with the National Institutes of Health Guide for the Care and Use of Laboratory Animals and the protocol approved by the Institutional Animal Care and Use Committee at Augusta University. The floxed ADK (ADK^flox/flox^) mice were generated by Xenogen Biosciences Corporation (Cranbury, NJ, USA). As shown in Supplementary Fig. 1b, Exon 7 of the *Adk* gene was flanked with *lox*P sites for conditional gene targeting. Cell-specific inactivation of ADK in endothelial cells (ADK^VEC-KO^) was achieved by cross-breeding Cdh5-Cre transgenic mice (The Jackson Laboratory) with ADK^flox/flox^ mice, all on a C57BL/6 background. For the atherosclerosis study, ADK^VEC-KO^ mice were bred with ApoE^−/−^ (The Jackson Laboratory) mice to generate ApoE^−/−^ADK^flox/flox^Cdh5^Cre/+^ (ApoE^−/−^/ADK^VEC-KO^) mice and their littermate control ApoE^−/−^ADK^flox/flox^ (ApoE^−/−^/ADK^WT^) mice. In all experiments, littermates from the same breeding pair were used as controls.

### Isolation of primary MAECs

Primary MAECs were isolated following a previously described protocol except that Matrigel was replaced with collagen gel^41^. The details were as follows:A. Preparation of collagen gel: the collagen gel was prepared by diluting type I collagen (BD Bioscience, San Jose, CA, USA) with endothelial growth medium 2 (EGM-2; Lonza, Basel, Switzerland) to a final concentration of 1.75 mg/ml. The collagen gel was added to 24-well plates (0.5 ml per well) and allowed to solidify at 37 °C for at least 30 min.

B. Preparation of aortic rings: 7-week-old male mice were used for MAEC isolation. Briefly, each mouse was sacrificed by CO_2_ asphyxiation and cleaned using 70% ethanol. The abdominal and thoracic cavities were opened, and the mouse was perfused with 3 ml PBS via the left ventricle. After perivascular fat and adventitia were removed from the ventral side of the aorta, the aortas were dissected out, rinsed five times with fresh PBS, and placed in a sterile dish of cold PBS. The aortas were then cut into small rings (~ 1 mm length), and each aortic ring was opened and carefully laid on a collagen gel with the endothelium directly facing the gel.

C. Thirty-six hours after tissue placement, the gel and aortic piece were kept hydrated with EGM-2. Care was taken to avoid completely submerging and dislodging the aorta piece from the gel. The explants were cultured at 37 °C and 5% CO_2_ in an incubator and monitored every day. When collection of aortic endothelial cells was needed, the medium was aspirated and the aortic segments were removed after visible cellular outgrowth from the aortic segments. To collect the MAECs, the collagen gel was digested by 0.3% collagenase H solution in PBS.

### Cell culture and treatments

HUVECs (ATCC, Manassas, VA, USA), at passage 3–8, and MAECs, at passage 2–4, were cultured in endothelial growth medium 2 (EGM-2; Lonza, Basel, Switzerland). In some experiments, 10 ng/ml TNF-α (R & D Systems, Minneapolis, MN, USA), 10 ng/ml IL-1β (R & D Systems, Minneapolis, MN, USA), 0–100 µM adenosine (Sigma, St Louis, MO, USA), 10 μM ITU (Tocris Bioscience, Bristol, United Kingdom), 2 µM ABT702 (Tocris Bioscience, Bristol, United Kingdom), 5 μM CGS21680 (Tocris Bioscience, Bristol, United Kingdom), 5 μM NECA (Tocris Bioscience, Bristol, United Kingdom), 5 μM MRS1754 (Tocris Bioscience, Bristol, United Kingdom), 5 μM ZM241385 (Tocris Bioscience, Bristol, United Kingdom), 10 μM nitrobenzylthioinosine (NBMPR) (Tocris Bioscience, Bristol, United Kingdom), 0.1–1mM inosine (Sigma, St Louis, MO, USA), 2 mM 5′-deoxy-5′ (methylthio)adenosine (MTA) (Sigma, St Louis, MO, USA), 2 μM 5-Aza-2′-deoxycytidine (Sigma, St Louis, MO, USA), 1 mM SAH (Sigma, St. Louis, MO, USA) or 20 µM adenosine-2′,3′-dialdehyde (Adox, Sigma, St Louis, MO, USA) was added to the culture medium. Human monocytic THP-1 cells (ATCC, Manassas, VA, USA) used for monocyte adhesion assay were cultured in RPMI medium 1640 (Thermo Scientific, Grand Island, NY, USA) containing 10% fetal bovine serum (FBS; Thermo Scientific, Grand Island, NY, USA).

### Adenoviral transduction of HUVECs

The green fluorescent protein (GFP)-labeled-ADK shRNA adenovirus targeting the 3′ UTR sequence of human ADK and the control adenovirus were generated by Vector Biolabs (Malvern, PA, USA). The recombinant adenovirus vector encoding His-fused human SAHH (NM_000687) and adenovirus vector containing GFP as a vector control were also generated by Vector Biolabs (Malvern, PA, USA). These adenoviruses were expanded inside HEK293 cells, and the virus concentration was determined using an Adeno-XTM rapid titer kit (Clontech, Mountain View, USA). HUVECs at 80% confluence were infected with the adenovirus (10 pfu/cell) and were used for experiments 36 h following the transduction.

### In vitro knockdown of A_2A_R, A_2B_R, and WDR5 using siRNAs

The A_2A_R (sc-39850), A_2B_R (sc-29642), WDR5 (sc-61798) and the control (sc-37007) siRNAs were purchased from Santa Cruz Biotechnology (Dallas, Texas, USA). Transfection of HUVECs was facilitated by siRNA transfection reagent (Santa Cruz Biotechnology, Dallas, Texas, USA) per the siRNA transfection protocol provided online by Santa Cruz Biotechnology. Six hours after the transfection, the transfection mixture was removed and replaced with EGM2 (Lonza, Basel, Switzerland). For double knockdown of A_2A_R, A_2B_R, or WDR5 and ADK in some experiments, just after the addition of fresh EGM2, HUVECs were transduced with the adenovirus containing shADK or its control.

### Monocyte adhesion assay

HUVECs (1 × 10^5^ cells) were seeded in 12-well flat-bottom plates for 12 h, followed by TNF-α treatment for 4 h. After removing TNF-α by completely washing with PBS, 5 × 10^5^ THP-1 cells were added to HUVECs. The cells were allowed to adhere at 37 °C for 30 min in fresh medium. The non-adherent cells were removed by washing three times with PBS. The number of adherent cells was counted after 4% paraformaldehyde treatment. Five random fields per well were captured for quantification. Adherent cells were marked as green and then counted by Image-Pro Plus version 5.0 software (Media Cybernetics, Inc., MD).

### Protein extraction and western blotting

HUVECs were lysed with a RIPA buffer (Sigma, St Louis, MO, USA) with 1% proteinase inhibitor cocktail (Roche, Basel, Switzerland) and 1% PMSF. After centrifugation of the cell lysates, proteins were quantified with a BCA assay and then loaded in 8–12% sodium dodecyl sulfate polyacrylamide gel electrophoresis gels at 20 μg per lane. Antibodies used in this study were as follows: anti-E-selectin (1:400, sc-14011, Santa Cruz Biotechnology, Dallas, Texas, USA), anti-ICAM-1 (1:400, sc-8439, Santa Cruz Biotechnology, Dallas, Texas, USA), anti-VCAM-1 (1:400, sc-1504, Santa Cruz Biotechnology, Dallas, Texas, USA) (1:1000, 13,662, Cell Signaling Technology, Danvers, MA, USA), anti-ADK (1:1000, ab38010, Abcam, Cambridge, MA, USA), anti-histone H3K4me2 (1:1000, 39,141, Active Motif, Carlsbad, CA, USA), anti-histone H3K4me3 (1:1000, 39,159, Active Motif, Carlsbad, CA, USA), anti-histone H3K9me2 (1:1000, 39,239, Active Motif, Carlsbad, CA, USA), anti-histone H3K27me2 (1:1000, 39,245, Active Motif, Carlsbad, CA, USA), anti-histone H3K36me2 (1:1000, 39,255, Active Motif, Carlsbad, CA, USA), anti-SAHH (1:200, sc-292967, Santa Cruz Biotechnology, Dallas, Texas, USA), anti-WDR5 (1:200, sc-393080, Santa Cruz Biotechnology, Dallas, Texas, USA), and β-actin (1:2000, 3700, Cell Signaling Technology, Danvers, MA, USA). Images were taken with the ChemiDoc MP system (Bio-Rad, Hercules, CA, USA), and band densities were quantified using Image Lab software (Bio-Rad, Hercules, CA, USA). See Supplementary Figs. 11–26 for gel source data.

### Quantitative real-time RT-PCR (qRT-PCR) analysis

The total RNA from HUVECs or from mice aortas was extracted with an RNeasy Mini Kit (Qiagen, Venlo, Netherlands), and qRT-PCR was performed as described previously^42^. Briefly, a 0.5–1 μg sample of RNA was used as a template for reverse transcription using the iScriptTM cDNA synthesis kit (Bio-Rad). qRT-PCR was performed on an ABI 7500 Real Time PCR System (Applied Biosystems) with the respective gene-specific primers listed in Supplemental Table 1. All qRT-PCR experiments were performed in biological triplicates that were repeated at least twice independently. Relative gene expression was converted using the 2^−ΔΔct^ method against the internal control β-actin. The data from each of these experimental groups were analyzed independently and revealed statistically significant differences between the indicated groups. Due to inherent variation in experiments performed at significantly different times, we limited the statistical analysis to data sets comprised of biological triplicates from one experiment performed at the same time.

### Measurement of intracellular adenosine

We used reverse-phase high-performance liquid chromatography (HPLC) to measure adenosine levels of cultured cells and collected tissues. Briefly, we separated supernatants of HUVECs, MAECs, or tissue lysates with protein concentration at 1 mg/ml with a C18 reverse-phase analytical column (250 mm × 4.6 mm I.D., 5 μm particle) (Aglient, Santa Clara, CA, USA). There are two solvents in the mobile phase. The first solution, solvent A, is a solution of 0.031 M Na_2_HPO_4_·2H_2_O and 0.068 M NaH_2_PO_4_·2H_2_O adjusted to pH 6.3. The second solution, solvent B is a solution of 100% methanol. We filtered solvent A through a 0.2-μm membrane filter prior to use in the assay. Then, we equilibrated the HPLC column with 80% solvent A and 20% solvent B and held the column constant at the equilibration conditions for 10 min. We set the flow-rate at 1 ml/min, and detection monitored at 254 nm. To detect adenosine, we applied a 20-μl aliquot of the acid extract of indicated samples directly onto the HPLC column. We determined the identity of adenosine by comparing retention times to an adenosine standard and further confirmed with enzymatic peak shift analysis. The values (µm/mg) shown in the results indicate the adenosine concentrations (uMol/l) in the cell/tissue lysates with protein concentration at 1 mg/ml.

### Measurement of intracellular cAMP

The cAMP concentrations were determined using a Cyclic AMP XP® assay kit (Cell Signaling Technology, Danvers, MA, USA) according to the manufacturer’s protocol.

### Measurement of intracellular homocysteine (Hcy)

The Hcy levels were determined by commercial Hcy ELISA Kit (Cell Biolabs, San Diego, CA, USA) according to the manufacturer’s protocol.

### S-adenosylhomocysteine hydrolase (SAHH) activity assay

SAHH activity was assessed using the adenosylhomocysteinase activity fluorometric assay kit (BioVision Incorporated, Milpitas, CA, USA) per the manufacturer’s instructions.

### Chromatin immunoprecipitation (ChIP) assay

ChIP assay was carried out with a ChIP-IT Express Enzymatic kit (Active Motif, Carlsbad, CA, USA). Briefly, HUVECs were treated with 1% formaldehyde for 10 min at room temperature. After quenching the formaldehyde crosslinking reaction with glycine stop-fix solution, the fixed HUVECs were washed with PBS. The HUVECs were suspended in lysis buffer, and the DNA enzymatically sheared to an average fragment size of 200–1000 bp. Solubilized chromatin was clarified by centrifugation for 10 min at 15,000×*g*rpm at 4 °C. The supernatant was quantified and incubated with protein G magnetic beads and anti-histone H3K4me2 (39,141, Active Motif, Carlsbad, CA, USA) and anti-histone H3K4me3 (39,159, Active Motif, Carlsbad, CA, USA) or pre-immune IgG overnight at 4 °C. Immune complex bound beads were washed with ChIP buffers, and then the chromatin was eluted with the elution buffer. After the crosslinks were reversed, the chromatin was treated with proteinase K for 1 h at 37 °C. Precipitated genomic DNA was amplified by RT-PCR with primers listed in Supplemental Table 1.

### Immunoprecipitation (IP) assay

HUVECs were washed twice with PBS and then were lysed with a RIPA buffer (Sigma, St. Louis, MO, USA) with 1% proteinase inhibitor cocktail (Roche, Basel, Switzerland) and 1% PMSF. After centrifugation of the cell lysates, the supernatant were preincubated for 1 h at 4 °C with 30 µl of protein G-sepharose beads (Sigma, St Louis, MO, USA) and then centrifuged to remove proteins that adhered nonspecifically to the beads and to obtain the target supernatant for the following IP experiment. Protein G-sepharose beads were incubated with anti-ADK (1.5 µg per 500 µg of total protein) (sc-23360, Santa Cruz Biotechnology, Dallas, Texas, USA), anti-SAHH (1.5 µg per 500 µg of total protein) (sc-292967, Santa Cruz Biotechnology, Dallas, Texas, USA) or anti-His (1:200, 2366, Cell Signaling Technology, Danvers, MA, USA) for 3–4 h. The antibody-conjugated protein G-sepharose beads and the target supernatant were incubated overnight. Immune complexes were isolated by centrifugation, washed 4 times with 0.05 M HEPES buffer, pH 7.1, containing 0.15% Triton X-100, 0.15 M NaCl, and 0.1 × 10-3 M sodium orthovanadate, and bound proteins were eluted by heating at 100 °C in loading buffer. Proteins were evaluated by immunoblotting.

### TNF-α treatment of mice

Two-month-old male ADK^VEC-KO^ mice and their wild-type (WT) controls were treated with a single intraperitoneal injection of recombinant murine TNF-α (10 μg/kg; R&D Systems, Minneapolis, MN). Five hours later, the thoracic aortas were isolated for RT-PCR analysis of E-selectin, ICAM-1 and VCAM-1 or for immunohistochemical (IHC) staining of ICAM-1 and VCAM-1 or for immunofluorescent (IF) staining of H3K4me2.

### Intravital microscopy

Intravital microscopy of postcapillary venules of the mouse cremaster muscle was used to study leukocyte rolling under different inflammatory conditions^43^. Each group includes four male mice at 8–12-weeks-old. Each mouse was anaesthetized with ketamine (125 mg/kg) and xylazine (12.5 mg/kg). Thereafter, the scrotum was opened, the cremaster muscle exteriorized, spread over a cover glass and superfused with warmed (35 ^o^C) bicarbonate-buffered saline. Intravital microscopy was performed on an upright microscope (Axioskop; Carl Zeiss, Goettingen, Germany) with a 40× saline immersion objective, NA 0.75. Movies were obtained using a digital camera (AxioCam MRm, Carl Zeiss) and analyzed using digital video software (ZEN 2012; Carl Zeiss). To induce leucocyte adhesion on endothelium, 10 μg/kg recombinant murine TNF-α (R&D Systems, Minneapolis, MN) was injected into mice by IP 4 h before opening the scrotum. One cremaster muscle for each mouse and 4 cremaster muscles total for each group were examined. For each cremaster, 2–3 different postcapillary venules and 3 different segments for one particular venule were recorded. Twenty-four to 36 movies for each group were analyzed to obtain data on leukocyte rolling velocity, rolling flux and leukocyte adhesion. Rolling velocity was calculated by measuring the distance covered by rolling cells with Image J, divided by the time of rolling and is given as µm/s. 5–10 randomly selected rolling leukocytes per venule (segment) and at least 60 rolling leukocytes in movies of one group were analyzed to obtain the averaged rolling velocities. Rolling flux was measured as the number of cells that roll past a line perpendicular to the vessel axis per 30 s. Leukocyte adhesion was defined as leukocyte adhesion longer than 30 s and expressed as cells per surface area. Surface area was calculated for each vessel by *S* = π*d*l_V_ where *d* is the diameter and *l*_v_ is the length of the vessel.

### Preparation of mouse aortas and quantification of atherosclerosis

To induce atherosclerosis, male ApoE^−/−^/ADK^VEC-KO^ or ApoE^−/−^/ADK^WT^ mice at 6 weeks of age were fed the western diet for 12 weeks. Aortas of atherosclerotic mice were collected, and both en face preparations of whole aortas and cross-sections of aortic sinuses were processed for Oil Red O staining. Each section of aortic sinus is 8 µM thick and two sections taken for examination were 48 µm apart. Therefore, six slides over a length of 288 µm of aortic sinus were analyzed. For each group, six mice were included. Images obtained from the above experiments were scanned into a Macintosh computer and analyzed with Image-Pro Plus version 5.0 software (Media Cybernetics, Inc., MD). The analysis of the en face Oil Red O staining was performed in a blinded manner.

### Focal cerebral ischemia–reperfusion (I/R)

The cerebral ischemic model was induced by occlusion of the middle cerebral artery on the right side as described previously^44^. Briefly, male mice at 8 weeks of age were anesthetized with 5% isoflurane (in 70% N_2_O, 30% O_2_) for induction and then with 1.5% isoflurane for maintenance. The rectal temperature was maintained at 37 ^o^C. After the right common carotid artery, internal carotid artery, and external carotid artery were surgically exposed, a coated 6–0 filament (6023PK, Doccol, Redlands, CA, USA) was inserted into the internal carotid artery through the external carotid artery stump and gently advanced 11 mm past the carotid bifurcation to occlude the middle cerebral artery. Thirty minutes after ischemia, the filament was gently withdrawn for reperfusion. The collar suture at the base of the external carotid artery stump was tightened. The skin was closed, anesthesia discontinued, and the animals were allowed to recover in the prewarmed cages. Mice were excluded from further experiments when excessive blood losses occurred during surgery, the operation time exceeded 90 min, mice did not recover from anesthesia within 15 min, or brain hemorrhage occurred during postmortem examination.

### Neurological characterization

Before euthanasia, a neuroscore assessment was conducted for each mouse (rating scale: 0 = no deficit, 1 = failure to extent left forepaw, 2 = decreased grip strength of left forepaw, 3 = circling to left by pulling the tail, and 4 = spontaneous circling). Each animal was scored for approximately 1 min, and assessment was repeated three times for consistency. All tests were evaluated in a blinded fashion with regard to the animal groups.

### Examination of infarct size

Twenty-four hours after cerebral I/R, mice were sacrificed by CO_2_ asphyxiation. The brains were removed rapidly and frozen at −20 °C for 5 min. Coronal slices were prepared at 2 mm from the frontal tips, and sections were immersed in 2% 2,3,5-tripenyltetrazolium chloride (TTC) (Sigma–Aldrich, MO, CA, USA) at 37 ^o^C for 20 min. The presence or absence of infarction was evaluated in a blinded fashion with regard to the animal groups by examining TTC-stained sections for the areas on the side of infarction that did not stain by TTC. The infarct size was indicated as percentage area of the coronal section in the infracted hemisphere.

### Histological procedures

For ICAM-1 and VCAM-1 IHC staining on mice aortas, 5 μm sections were cut through the thoracic aortas following paraffin embedding. For Mac-2 IHC staining in aortic root, 5 μm frozen sections were cut through the aortic root in the heart. Sections were fixed in cold acetone, blocked with 0.1% triton X-100, normal serum of the same species as the secondary antibody, Avidin/Biotin and quenched with 3% hydrogen peroxide. After blocking, sections were incubated with anti-ICAM-1 (3 µg/ml, ab25375, Abcam, Cambridge, MA, USA), anti-VCAM-1 (3 µg/ml, 1510-01, Southern Biotech, Birmingham, AL, USA) and anti-Mac-2 (3 µg/ml, ACL8942F, Accurate Chemical & Scientific Corporation, Westbury, NY, USA). Biotinylated secondary antibodies were applied and VECTASTAIN® ABC reagents (1: 200, Vector Labs, Burlingame CA) were used according to the manufacturer’s instructions. Control sections were incubated with IgG2a isotype antibody (eBioscience). Sections were counterstained with hematoxylin. Hematoxylin and eosin (H&E) staining was performed to show the necrotic areas on cross-sections of atherosclerotic lesions. Quantification of % Mac-2 staining area and necrotic size was performed using the Image-Pro Plus version 5.0 software (Media Cybernetics, Inc., MD). Data are reported as a mean of eight sections of aortic root per heart.

For ICAM-1 and VCAM-1 IF staining in aortic root, 5 μm frozen sections were cut through the aortic root in the heart. For CD31 and H3K4me2 co-IF staining on mice aortas, 5 μm sections were cut through the thoracic aortas following paraffin embedding. Sections were heated at 98 °C for 8 min in citric acid buffer for antigen retrieval and incubated with anti-ICAM-1 (3 µg/ml, ab25375, Abcam, Cambridge, MA, USA), anti-VCAM-1 (3 µg/ml, 1510-01, Southern Biotech, Birmingham, AL, USA), anti-CD31 (3 µg/ml, Dianova, Hamburg, Germany) and anti-histone H3K4me2 (3 µg/ml, 39,141, Active Motif, Carlsbad, CA, USA) followed by incubation with an Alexa Fluor 594-labeled secondary antibody (1:400, Molecular Probes) and/or Alexa Fluor 488-labeled secondary antibody (1:400, Molecular Probes). The slides were then immersed in ProLong Gold mounting medium with DAPI (Invitrogen) to visualize the nuclei.

For Mac-2 and Iba-1 IHC staining in brain, free-floating sections encompassing the entire procerebrum were prepared by using a cryostat microtome (Leica Microsystems, Wetzlar, Germany). After fixing and blocking, sections were incubated overnight with anti-Mac-2 (3 µg/ml, ACL8942F, Accurate Chemical & Scientific Corporation, Westbury, NY, USA) and anti-Iba-1 (3 µg/ml, 019-19741, Wako Chemicals USA, Richmond, VA, USA). Biotinylated secondary antibodies were applied and VECTASTAIN® ABC reagents (1:200, Vector Labs, Burlingame CA) were used according to the manufacturer’s instructions. Sections were then mounted onto slides (Fisher Scientific, Pittsburgh, PA, USA) for counterstaining of hematoxylin. Four different areas of each section were observed.

### Flow cytometry analysis of immune cell populations

Twenty-four hours after cerebral I/R, mice were anesthetized with ketamine (125 mg/kg) and xylazine (12.5 mg/kg) and then perfused through the left ventricle with 10 ml of ice-cold PBS. The affected brain was collected and minced with Medimachine (BD Pharmingen, San Jose, CA, USA) in PBS with 1% FBS. Tissue suspensions were filtered through 50-µm filter (BD Pharmingen, San Jose, CA, USA) and then centrifuged at 300 g for 10 min at room temperature. Pellets were resuspended in 3 ml of 50% Percoll and overlaid on the top of a gradient containing 3 ml of 30% of Percoll. The gradient was centrifuged at 500×*g* for 20 min at room temperature. Cells were collected from the 30% to 50% interface and resuspended on 200 μl of 2.5% BSA in PBS with Fc Block reagent (BD Pharmingen, San Jose, CA, USA). Cell suspensions were incubated with anti-CD11b-FITC (1 µg/100 µl, 11-0112-82, eBioscience, San Diego, CA, USA), anti-F4/80-PE (0.5 µg/100 µl, 565410, BD Pharmingen, San Jose, CA, USA), anti-Ly6G-Percp-cy5.5 (0.4 µg/100 µl, 560602, BD Pharmingen, San Jose, CA, USA) or anti-CD45.2-APC (0.3 µg/100 µl, 558702, BD Pharmingen, San Jose, CA, USA) antibodies. Stained cells were washed and resuspended in 400 μl of FACS Flow (BD Pharmingen, San Jose, CA, USA), and the whole suspension was analyzed using a FACSCalibur flow cytometer with CellQuest software (BD Pharmingen, San Jose, CA, USA). Isotype controls (BD Pharmingen, San Jose, CA, USA) were used in parallel.

### Apoptosis assay

For HUVEC apoptosis assay, HUVECs were serum-starved for 8 h to remove the effect of any exogenous growth factors. Thereafter, cells were treated with 10 ng/ml TNF-α in the presence or absence of 100 µM adenosine for 6 h. Apoptotic cells (Annexin V^+^) were analyzed with flow cytometry. At least 10,000 events were collected. Data were analyzed with CellQuest v3.3 software (BD Bioscience, San Jose, CA, USA) as instructed.

For apoptosis assay in brain tissue sections, the terminal deoxynucleotidyl transferase-mediated dUTP biotin nick end-labeling (TUNEL) assay was performed with the DeadEnd colorimetric apoptosis detection kit (Promega, Madison, WI, USA).

### Microarray analysis

RNA from ADK knockdown HUVECs and their controls was extracted using RNeasy mini-kit (Qiagen, Venlo, Netherlands) following the manufacturer’s recommendations. RNA quality and integrity were evaluated with Fragment Analyzer (Advanced Analytical, Ankeny, IA, USA), and the samples with RQN > 9 were used for analysis (three of each group). The Integrated Genomics Shared Resource at the Augusta University performed sample preparation and hybridization on Affymetrix Human Gene 2.0 ST arrays (902136). Non-used mRNA was used for qPCR. Fold-change analysis was performed using the R package limma, and *P*-values were Benjamini-Hochberg (BH) adjusted. The array results are available in the NCBI’s Gene Expression Omnibus (GEO) database (Accession code: GSE101126).

### Statistical analysis

The minimum animal numbers and sample sizes required to achieve statistical significance were determined by power analysis and prior experience. Grouping was carried out in a randomized manner. The data are presented as the mean ± SEM and were analyzed by one-way analysis of variance (ANOVA) with Tukey’s post hoc test or Student’s *t*-test to evaluate two-tailed levels of significance. The null hypothesis was rejected at *P ≤ *0.05. All biological experiments were repeated at least three times using independent cell cultures or individual animals (biological replications).

### Data availability

All the data supporting the findings of this study are available in this published article and its Supplementary Information files, or are available from the corresponding author on reasonable request. The array results are available in the NCBI’s Gene Expression Omnibus (GEO) database (Accession code: GSE101126).

## Electronic supplementary material

Supplementary Information

Supplementary Movie 1

Supplementary Movie 2

Supplementary Movie 3

Supplementary Movie 4
