# Supplementary material for: Regulation of endothelial intracellular adenosine via adenosine kinase epigenetically modulates vascular inflammation
Source: Nat Commun. 2017 Oct 16;8:943. doi: 10.1038/s41467-017-00986-7 (PMC5643397; doi:10.1038/s41467-017-00986-7)
Supplement: Supplementary file 1 — Supplementary Information [file 41467_2017_986_MOESM1_ESM.pdf]

Description of Supplementary Files

File name: Supplementary Information
Description: Supplementary figures and supplementary table.

File name: Supplementary Movie 1
Description: Intravital microscopy showing leukocyte interactions in the cremaster muscle model of ADK^{WT} mice ($ADK^{f/f}$ mice). Cremaster muscle was exteriorized and super-perfused with physiological buffer. Intravital microscopy was performed as described in Supplemental Materials and Methods.

File name: Supplementary Movie 2
Description: Intravital microscopy showing leukocyte interactions in the cremaster muscle model of $ADK^{VEC\text{-}KO}$ mice ($ADK^{f/f}/Cdh5^{cre}$ mice,). Cremaster muscle was exteriorized and super-perfused with physiological buffer. Intravital microscopy was performed as described in Supplemental Materials and Methods.

File name: Supplementary Movie 3
Description: Intravital microscopy showing leukocyte interactions in the inflamed cremaster muscle model of ADK^{WT} mice ($ADK^{f/f}$ mice). Each ADK^{WT} mouse was intraperitoneally injected with murine TNF-α (10 µg/kg). 4 hours later, cremaster muscle was exteriorized and super-perfused with physiological buffer. Intravital microscopy was performed as described in Supplemental Materials and Methods.

File name: Supplementary Movie 4
Description: Intravital microscopy showing leukocyte interactions in the inflamed cremaster muscle model of $ADK^{VEC\text{-}KO}$ mice ($ADK^{f/f}/Cdh5^{cre}$ mice,). Each $ADK^{VEC\text{-}KO}$ mouse was intraperitoneally injected with murine TNF-α (10 µg/kg). 4 hours later, cremaster muscle was exteriorized and super-perfused with physiological buffer. Intravital microscopy was performed as described in Supplemental Materials and Methods.

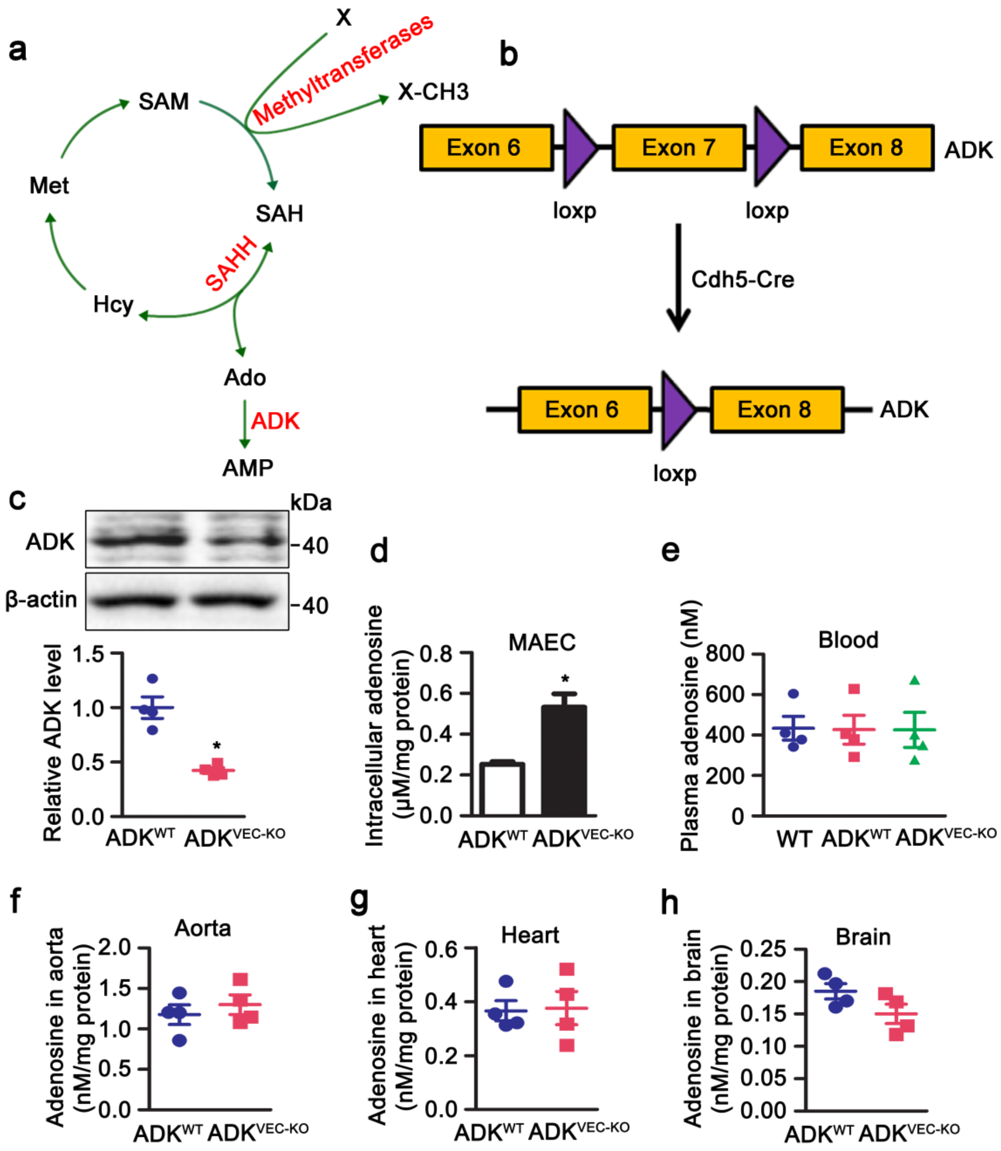

Supplementary Figure 1: Endothelial cell-specific deletion of ADK in ADK^{VEC-KO} mice and the measurement of intracellular adenosine. a, Biochemistry of the transmethylation reaction. SAM functions as a methyl-group donor in a wide range of transmethylation reactions. By providing the methyl group, SAM is converted into SAH, which is then hydrolyzed into Hcy and Ade. Hcy is subsequently remethylated through Met to SAM and Ade is metabolized into AMP by ADK. The thermodynamics of the SAHH reaction favors condensation of adenosine and Hcy to produce SAH; physiologically, SAH is hydrolyzed when adenosine and Hcy are removed, and increased levels of Ade therefore cause reversal of the reaction. Abbreviations are as follows: SAM: S-adenosylmethionine; SAH: S-adenosylhomocysteine; Hcy: Homocysteine; Ado:

Adenosine; Met, Methionine, AMP: Adenosine monophosphate; ADK: Adenosine kinase. **b,** Schematic of Cre-mediated endothelial-specific ADK exon-7 deletion. **c,** Western blot detection and densitometric quantification of ADK in mouse aortic endothelial cells from ADK^{WT} and $ADK^{VEC\text{-}KO}$ mice (n = 4). **d,** Quantification of intracellular adenosine in MAECs isolated from ADK^{WT} and $ADK^{VEC\text{-}KO}$ mice (n = 5). **e,** Quantification of adenosine level in plasma drawn from ADK^{WT} and $ADK^{VEC\text{-}KO}$ mice (n = 4 mice per group). **f-h,** Quantification of adenosine levels in aortas (**f**), hearts (**g**), and brains (**h**) from ADK^{WT} and $ADK^{VEC\text{-}KO}$ mice (n = 4 mice per group). For all bar graphs, data are the mean ± SEM, * $P < 0.05$ (Unpaired, two-tailed Student's *t*-test).

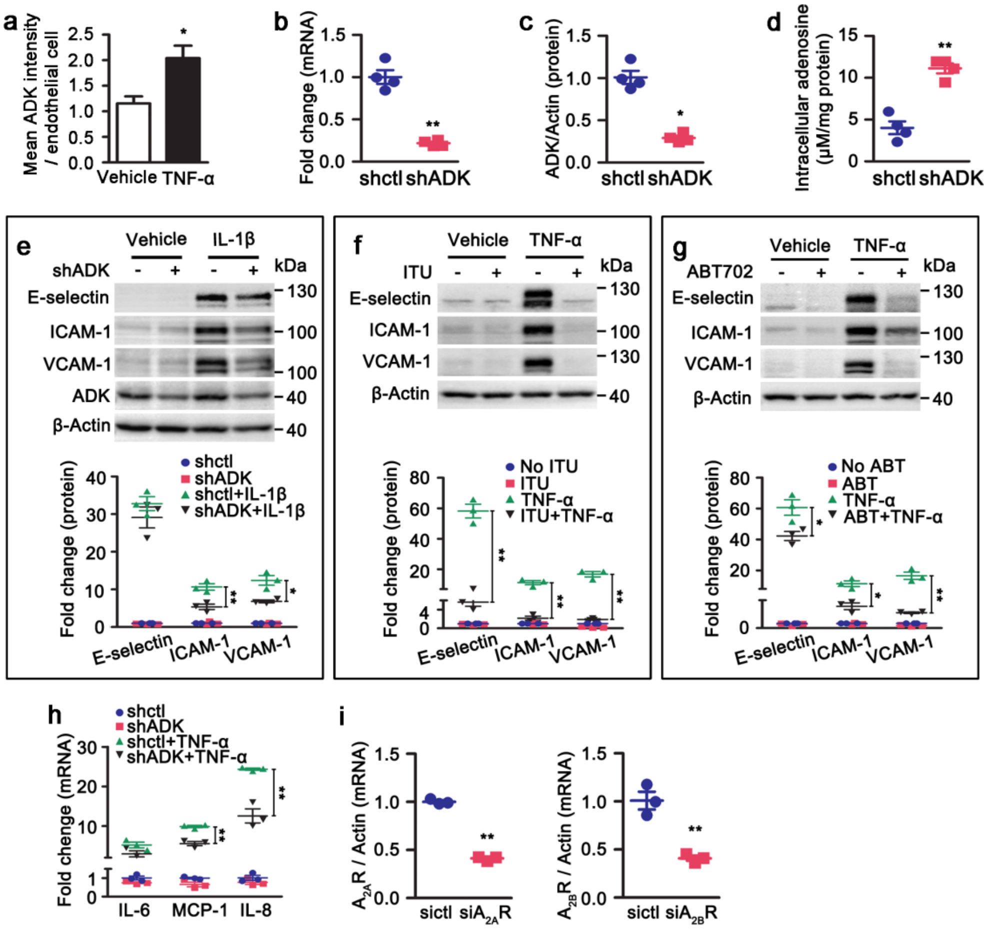

Supplementary Figure 2: The role of ADK inactivation in endothelial inflammation. a, Quantification of the mean fluorescence intensity for ADK expression per endothelial cell in aortas from WT mice treated with vehicle or TNF-α (10 ng per gram mouse body weight for 5 h) (n = 5-6 mice per group). **b,** Quantification of ADK mRNA levels in ADK KD or Ctrl HUVECs (n = 4). **c,** Quantification of ADK protein expression in ADK KD or Ctrl HUVECs by Western blot (n = 4). **d,** Quantification of intracellular adenosine in HUVECs. The level of intracellular adenosine was quantified by HPLC in HUVECs infected with adenoviral shctl or shADK for 36 hours (n = 4). **e,** Western blot detection and densitometric quantification of adhesion molecule expression in ADK KD or Ctrl HUVECs treated with IL-1β at 10ng/ml for 4 h (n = 3). **f,** Western blot detection and densitometric quantification of adhesion molecule expression in TNF-α (10ng/ml for 4 h)-treated HUVECs pretreated with 10μM ITU for 30 min (n = 3). **g,** Western blot detection and densitometric quantification of adhesion molecule expression in TNF-α (10ng/ml for 4 h)-treated HUVECs pretreated with 2μM ABT702 for 30 min (n = 3). **h,**

RT-PCR analysis of mRNA levels of IL-6, MCP-1 and IL-8 in TNF-α (10ng/ml for 2 h)-treated ADK KD or Ctrl HUVECs (n = 3). **i,** Quantification of the levels of $A_{2A}R$ and $A_{2B}R$ in HUVECs transfected with control, $A_{2A}R$, or $A_{2B}R$ siRNA (n = 3). All images are representative. For all bar graphs, data are the mean ± SEM, * $P < 0.05$ and ** $P < 0.01$ (Unpaired, two tailed Student's *t*-test).

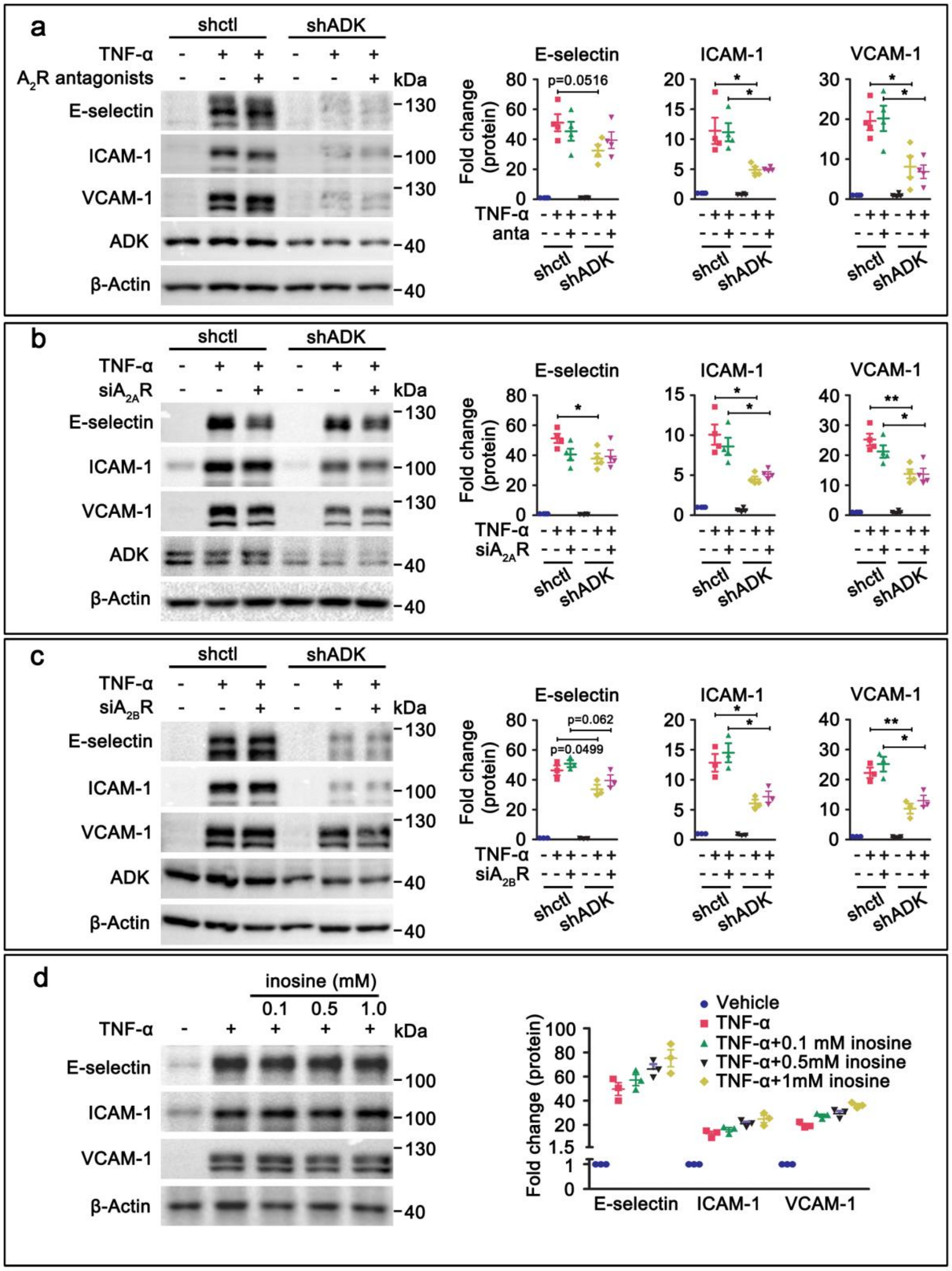

Supplementary Figure 3: The role of adenosine receptors in ADK KD-induced suppression of endothelial inflammation. **a,** Western blot detection and densitometric quantification of adhesion molecules in TNF-α (10ng/ml for 4 h)-treated ADK KD or Ctrl HUVECs preincubated with 5μM ZM 241385 and 5μM MRS 1754 for 30 min (n = 4). **b,** Western blot detection and densitometric quantification of adhesion molecule expression in TNF-α (10ng/ml for 4 h)-treated

ADK KD or Ctrl HUVECs transiently transfected with control or $A_{2A}R$ siRNA (n = 4). **c,** Western blot detection and densitometric quantification of adhesion molecule expression in TNF-α (10ng/ml for 4 h)-treated ADK KD or Ctrl HUVECs transiently transfected with control or $A_{2B}R$ siRNA (n = 3). **d,** Western blot detection and densitometric quantification of adhesion molecule expression in TNF-α (10ng/ml for 4 h)-treated HUVECs pretreated with inosine for 30 min at concentration range of 0.1-1mM (n = 3). All images are representative. For all bar graphs, data are the mean ± SEM, * $P < 0.05$ and ** $P < 0.01$ (One-way ANOVA with Tukey's *post hoc* test).

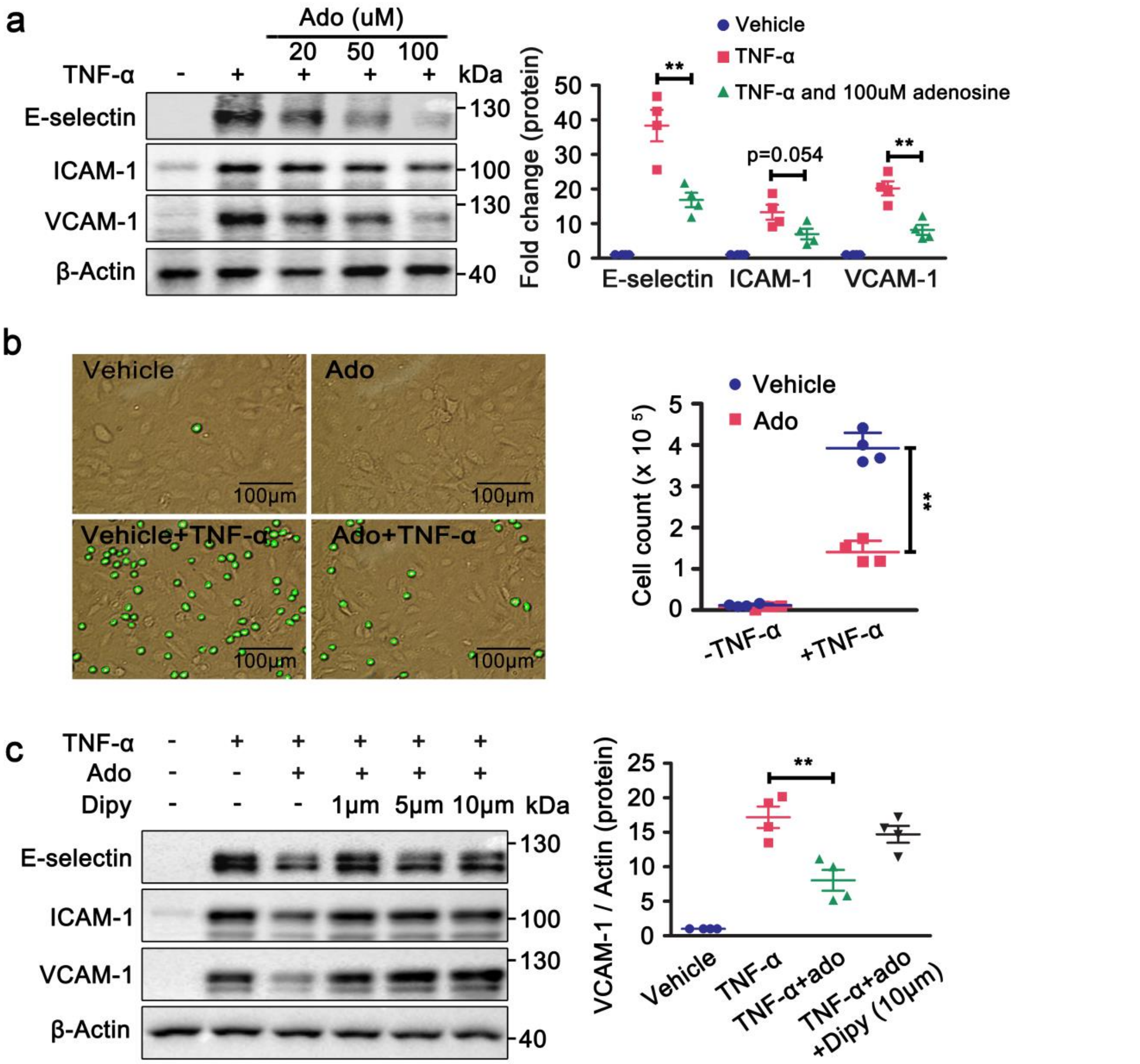

Supplementary Figure 4: The role of intracellular adenosine in endothelial inflammation. a, Western blot detection and densitometric quantification of adhesion molecule expression in TNF-α (10ng/ml for 4 h)-treated HUVECs pretreated with adenosine for 30 min at concentration range of 20-100μM (n = 4). **b,** Representative images and quantification of monocyte adhesion on TNF-α (10ng/ml for 4 h)-treated HUVECs pretreated with 100μM adenosine for 30 min (scale bar, 100μm; n = 4). **c,** Western blot detection and densitometric quantification of adhesion molecule expression in HUVECs. HUVECs, pretreated for 30 min with 5 or 10μM dipyridamole, were incubated with 100μM adenosine for 30 min and then stimulated with TNF-α at 10ng/ml for 4 h (n = 4). All images are representative. For all bar graphs, data are the mean ± SEM, ** $P < 0.01$ (Unpaired, two-tailed Student's *t*-test).

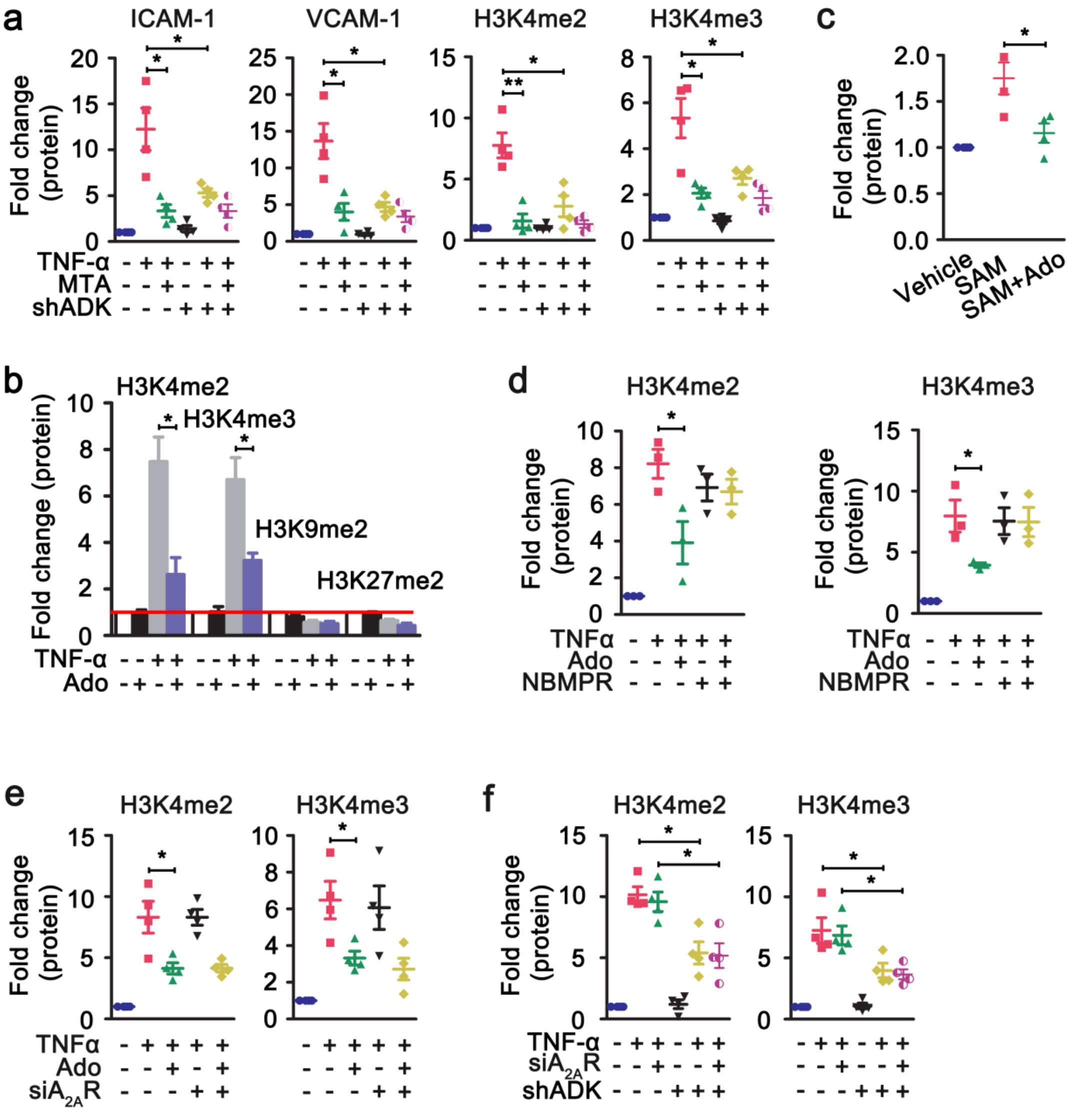

Supplementary Figure 5: The effect of ADK on the methylation of H3K4. a, Quantification of adhesion molecule expression and H3K4 methylation in TNF-α (10ng/ml for 4 h)-treated ADK KD or Ctrl HUVECs pretreated with 2 mM MTA for 30 min (n = 4). **b,** Quantification of H3K4, H3K9 and H3K27 methylation in TNF-α (10ng/ml for 12 h)-treated HUVECs pretreated with 100 μM adenosine for 30 min (n = 5). **c,** Quantification of H3K4me2 in HUVEC whole protein lysate supplemented with 1mg/ml SAM or SAM together with 10μM adenosine for 60 min (n = 4). **d,** Quantification of H3K4 methylation in HUVECs. HUVECs, pretreated for 30 min with 10μM NBMPR, were incubated with 100μM adenosine for 30 min and then stimulated with TNF-α at 10ng/ml for 4 h (n = 3). **e,** Quantification of H3K4 methylation in TNF-α (10ng/ml for 4 h)-treated A_{2A}R KD or Ctrl HUVECs pretreated with 100 μM adenosine for 30

min (n = 4). **f,** Quantification of H3K4 methylation in TNF-α (10ng/ml for 4 h)-treated ADK KD or Ctrl HUVECs transiently transfected with control or $A_{2A}R$ siRNA (n = 4). For all bar graphs, data are the mean ± SEM, * $P < 0.05$ and ** $P < 0.01$ (One-way ANOVA with Tukey's *post hoc* test).

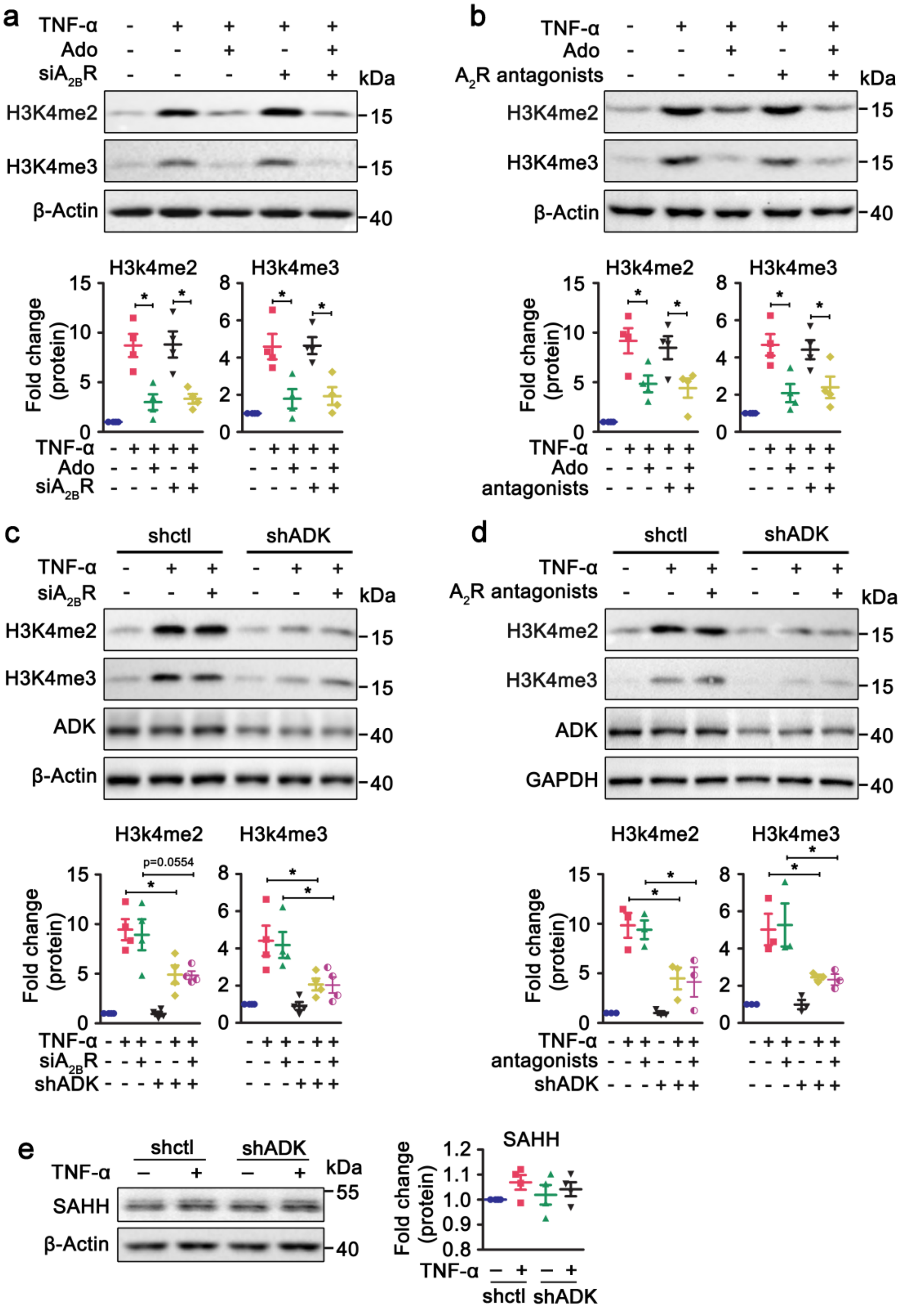

Supplementary Figure 6: The role of adenosine receptors in ADK KD-induced inhibition of H3K4 methylation. a, Western blot detection and densitometric quantification of H3K4 methylation in TNF-α (10ng/ml for 4 h)-treated A_{2B}R KD or Ctrl HUVECs pretreated with 100 μM adenosine for 30 min (n = 4). **b,** Western blot detection and densitometric quantification of H3K4 methylation in HUVECs. HUVECs, pretreated for 30 min with 5μM ZM 241385 and

5μM MRS 1754, were incubated with 100μM adenosine for 30 min and then stimulated with TNF-α at 10ng/ml for 4 h (n = 4). **c,** Western blot detection and densitometric quantification of H3K4 methylation in TNF-α (10ng/ml for 4 h)-treated ADK KD or Ctrl HUVECs transiently transfected with control or $A_{2B}R$ siRNA (n = 4). **d,** Western blot detection and densitometric quantification of H3K4 methylation in TNF-α (10ng/ml for 4 h)-treated ADK KD or Ctrl HUVECs preincubated with 5μM ZM 241385 and 5μM MRS 1754 for 30 min (n = 3). All images are representative. **e,** Western blot detection and densitometric quantification of SAHH in TNF-α (10ng/ml for 12 h)-treated ADK KD or Ctrl HUVECs (n = 4). For all bar graphs, data are the mean ± SEM, * $P < 0.05$ and ** $P < 0.01$ (One-way ANOVA with Tukey's *post hoc* test).

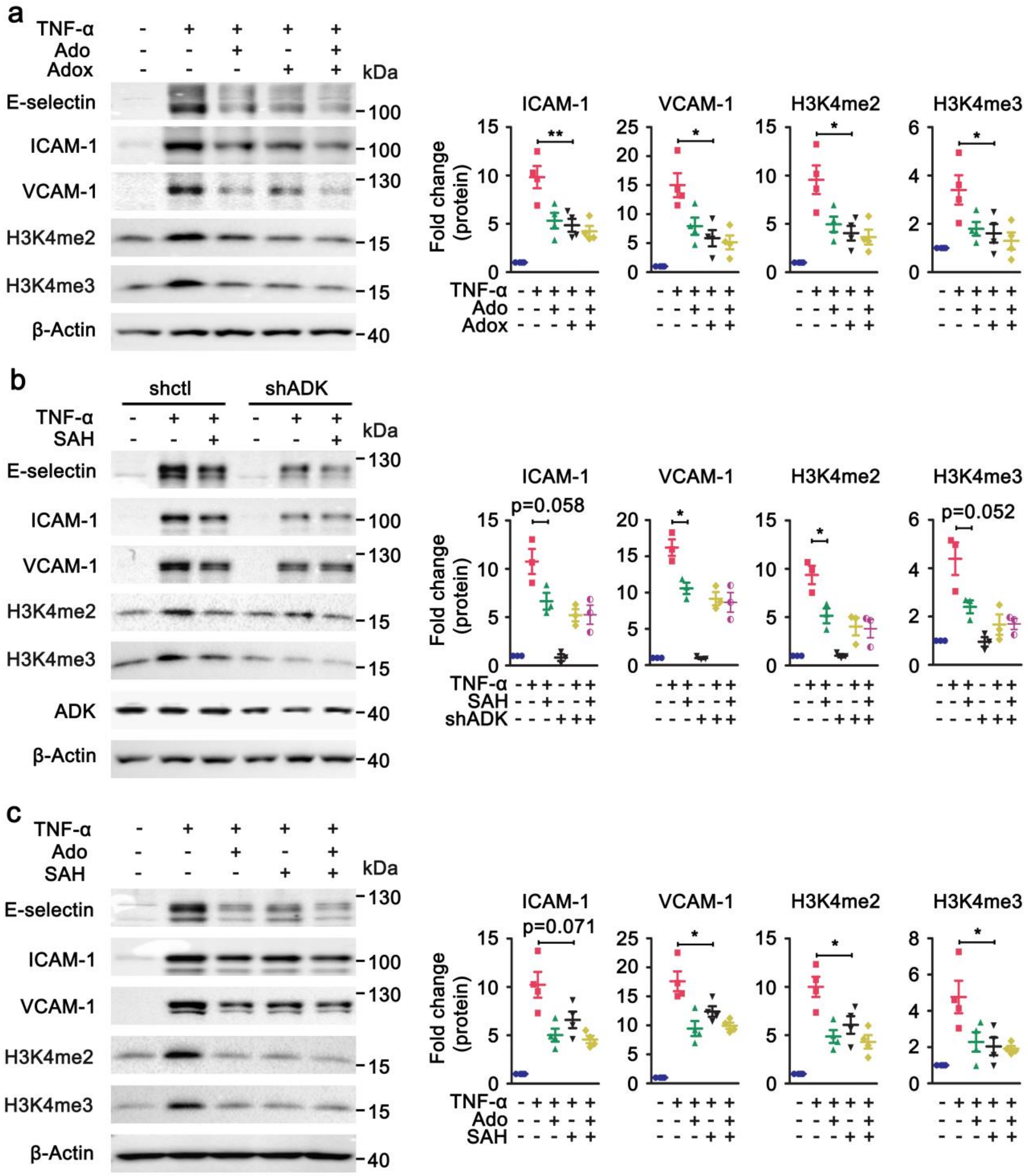

Supplementary Figure 7: The role of SAHH inactivation in ADK KD-induced suppression of endothelial inflammation. a, Western blot detection and densitometric quantification of adhesion molecule expression and H3K4 methylation in HUVECs. HUVECs, pretreated for 30 min with 20µM adenosine-2', 3'-dialdehyde (Adox), were incubated with 100µM adenosine for 30 min and then stimulated with TNF-α at 10ng/ml for 4 h (n =4). **b,** Western blot detection and densitometric quantification of adhesion molecule expression and H3K4 methylation in TNF-α (10ng/ml for 4 h)-treated ADK KD or Ctrl HUVECs preincubated with 1mM SAH for 20 h (n =

3). **c,** Western blot detection and densitometric quantification of adhesion molecules and H3K4 methylation in HUVECs. HUVECs, pretreated for 20 h with 1mM SAH were incubated with 100μM adenosine for 30 min and then stimulated with TNF-α at 10ng/ml for 4 h (n = 4). All images are representative. For all bar graphs, data are the mean ± SEM, * $P < 0.05$, ** $P < 0.01$ and # indicates no significance (One-way ANOVA with Tukey's *post hoc* test).

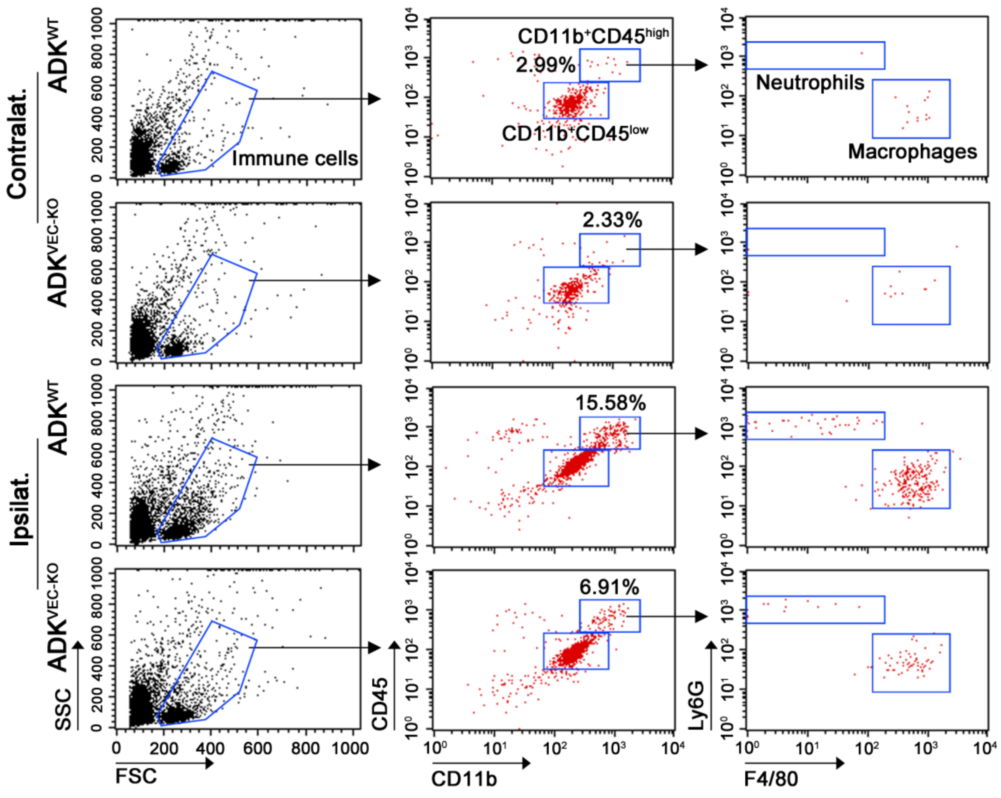

Supplementary Figure 8: The gating strategy of flow cytometry. The gating strategy for each inflammatory cell population indicating resident microglia as $CD11b^{+}CD45^{low}$, infiltrated leukocytes as $CD11b^{+}CD45^{high}$, $CD11b^{+}CD45^{high}F4/80^{+}$ as macrophages/activated microglia cells, and infiltrated neutrophils as $CD11b^{+}CD45^{high}Ly6G^{+}$.

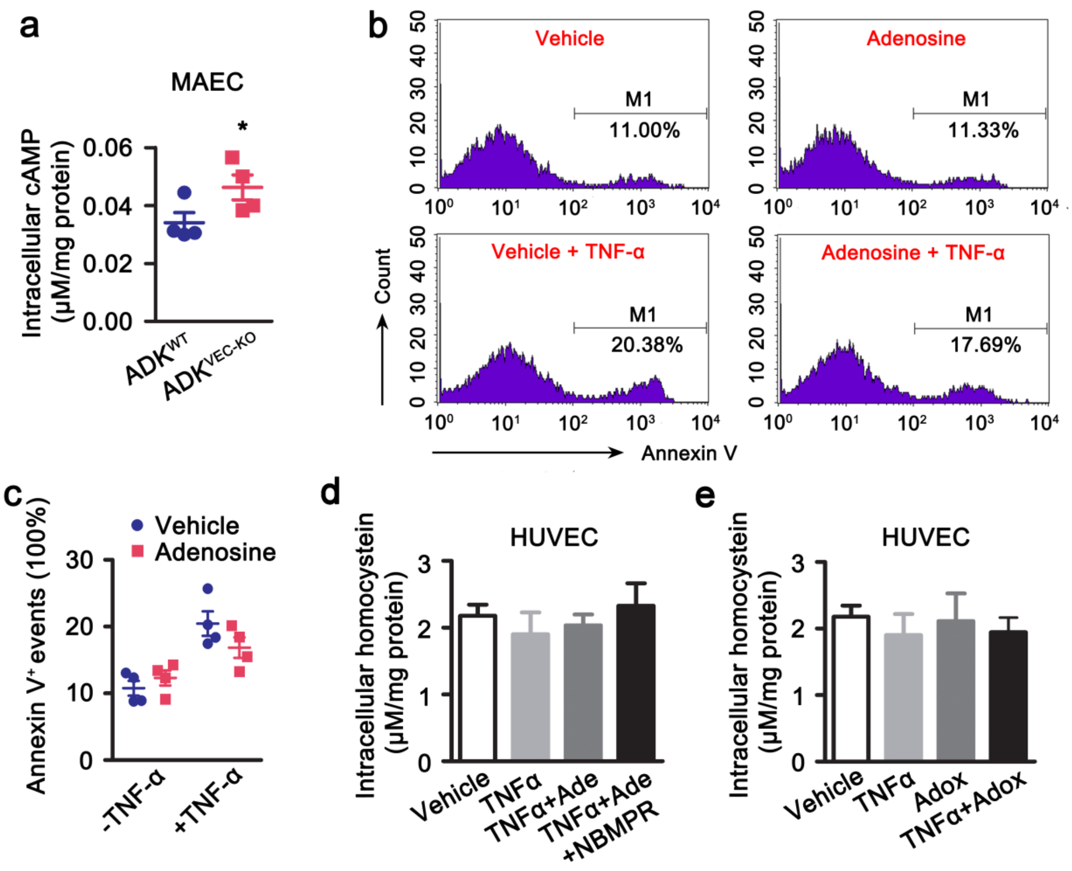

Supplementary Figure 9: The role of ADK KD in endothelial apoptosis and the measurement of intracellular adenosine as well as homocysteine. a, Quantification of the intracellular cAMP level in MAECs isolated from ADK^{WT} and ADK^{VEC-KO} mice (n = 4). **b,** Flow cytometry analysis of apoptosis in TNF-α (10ng/ml for 6 h)-treated HUVECs pretreated with 100 μM adenosine for 30 min (n = 4). **c,** Quantification of the Annexin V^+ HUVEC ratio for each group (n = 4). **d,** Quantification of intracellular Hcy levels in HUVECs. HUVECs, pretreated for 30 min with 10μM NBMPR, were incubated with 100μM adenosine for 30 min and then stimulated with TNF-α at 10ng/ml for 30 min (n = 5). **e,** Quantification of intracellular Hcy level in TNF-α (10ng/ml for 30min)-treated HUVECs pretreated with 20μM adenosine-2', 3'-dialdehyde (Adox) for 30 min (n = 5). For all bar graphs, data are the mean ± SEM, * $P < 0.05$ (Unpaired, two-tailed Student's *t*-test for a; one-way ANOVA with Tukey's *post hoc* test for c-e).

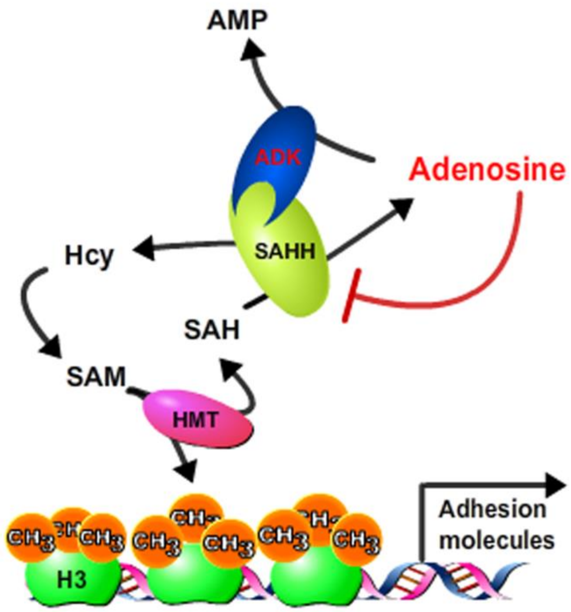

Supplementary Figure 10: Schematic of the proposed role of intracellular adenosine and ADK in endothelial inflammation.

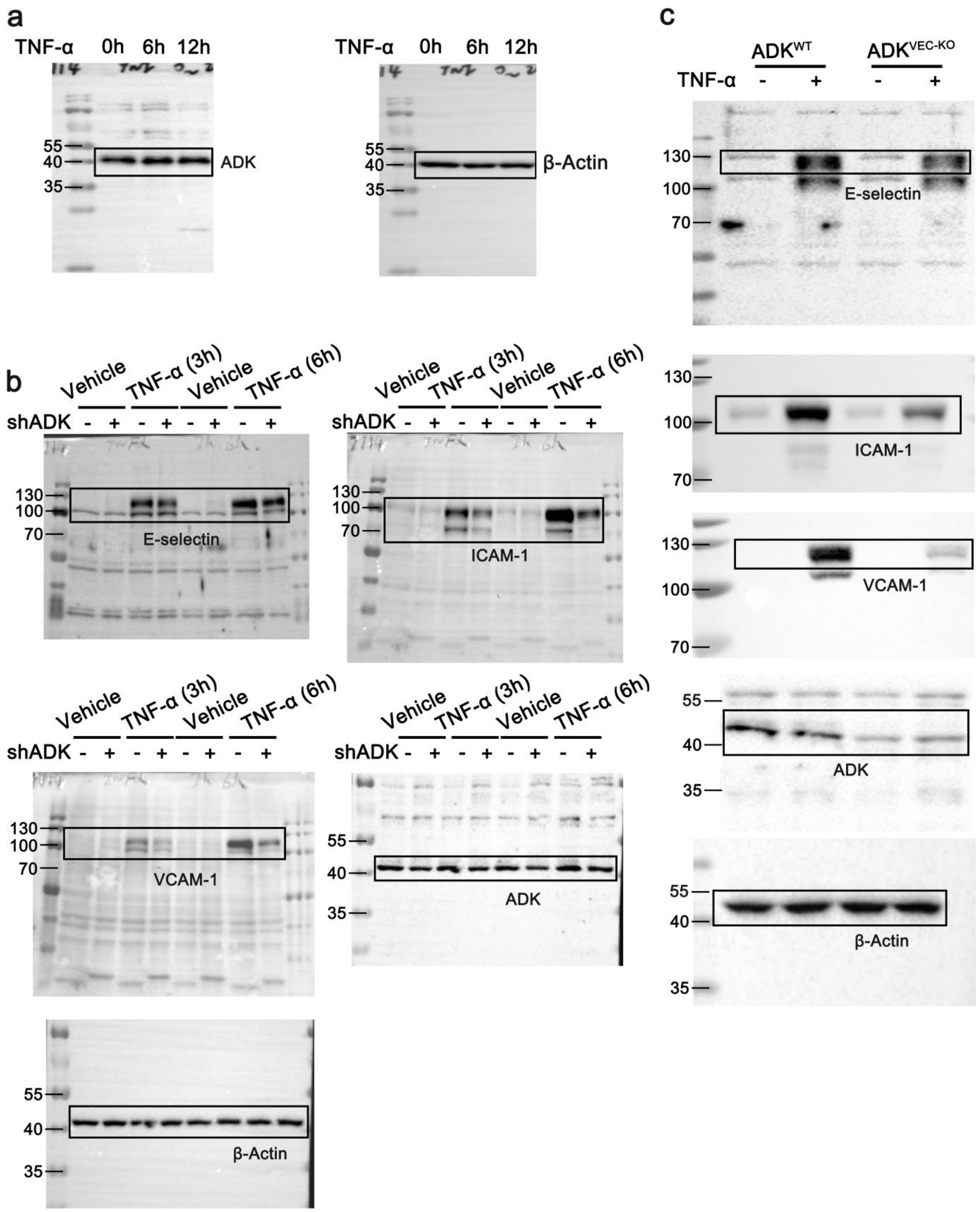

Supplementary Figure 11: Full gel scans for Fig. 1a, 1f and 1i. a, Gel scans for Fig. 1a. **b,** Gel scans for Fig. 1f. **c,** Gel scans for Fig. 1i.

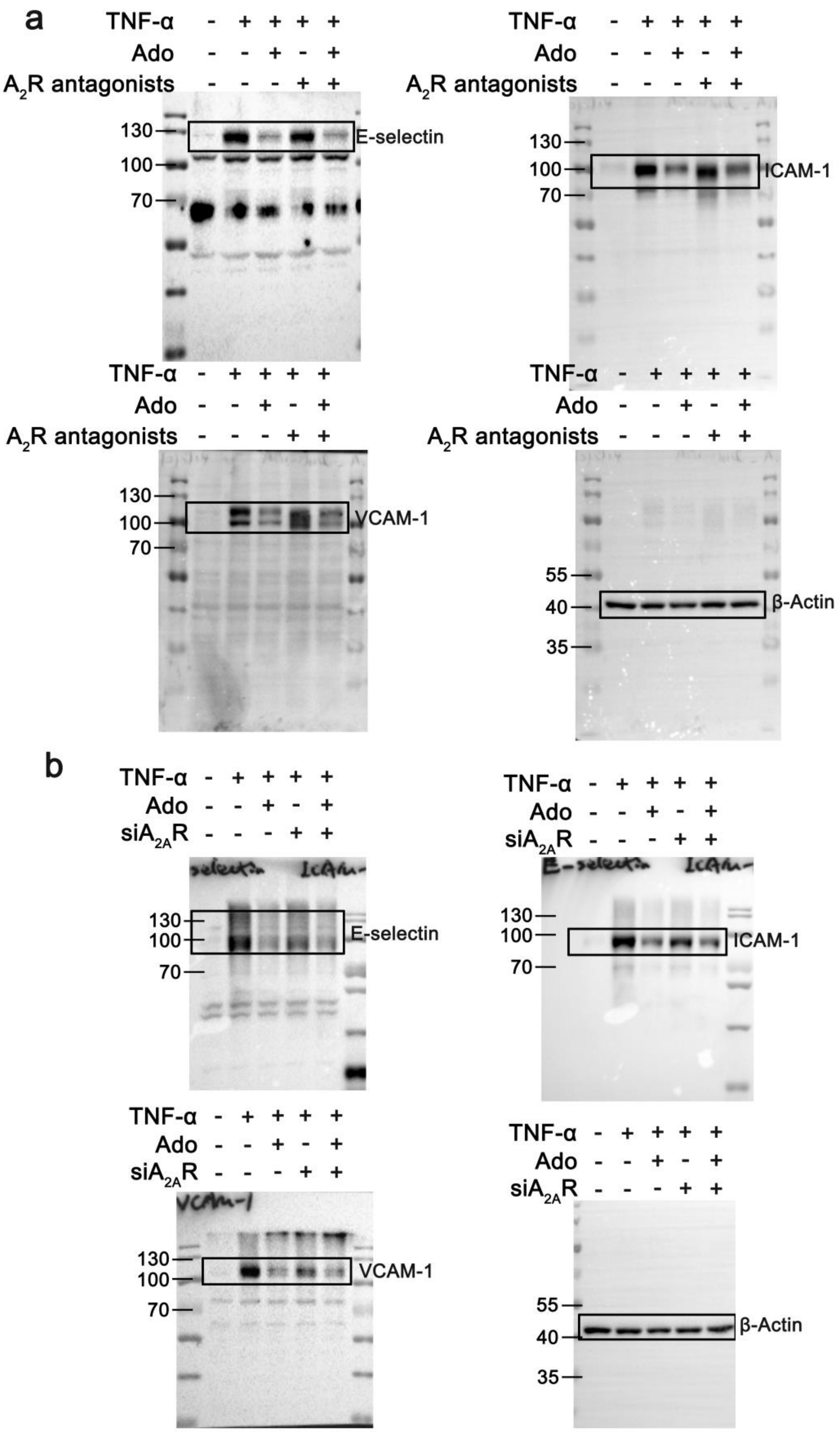

Supplementary Figure 12: Full gel scans for Fig. 2a and 2b. a, Gel scans for Fig. 2a. **b,** Gel scans for Fig. 2b.

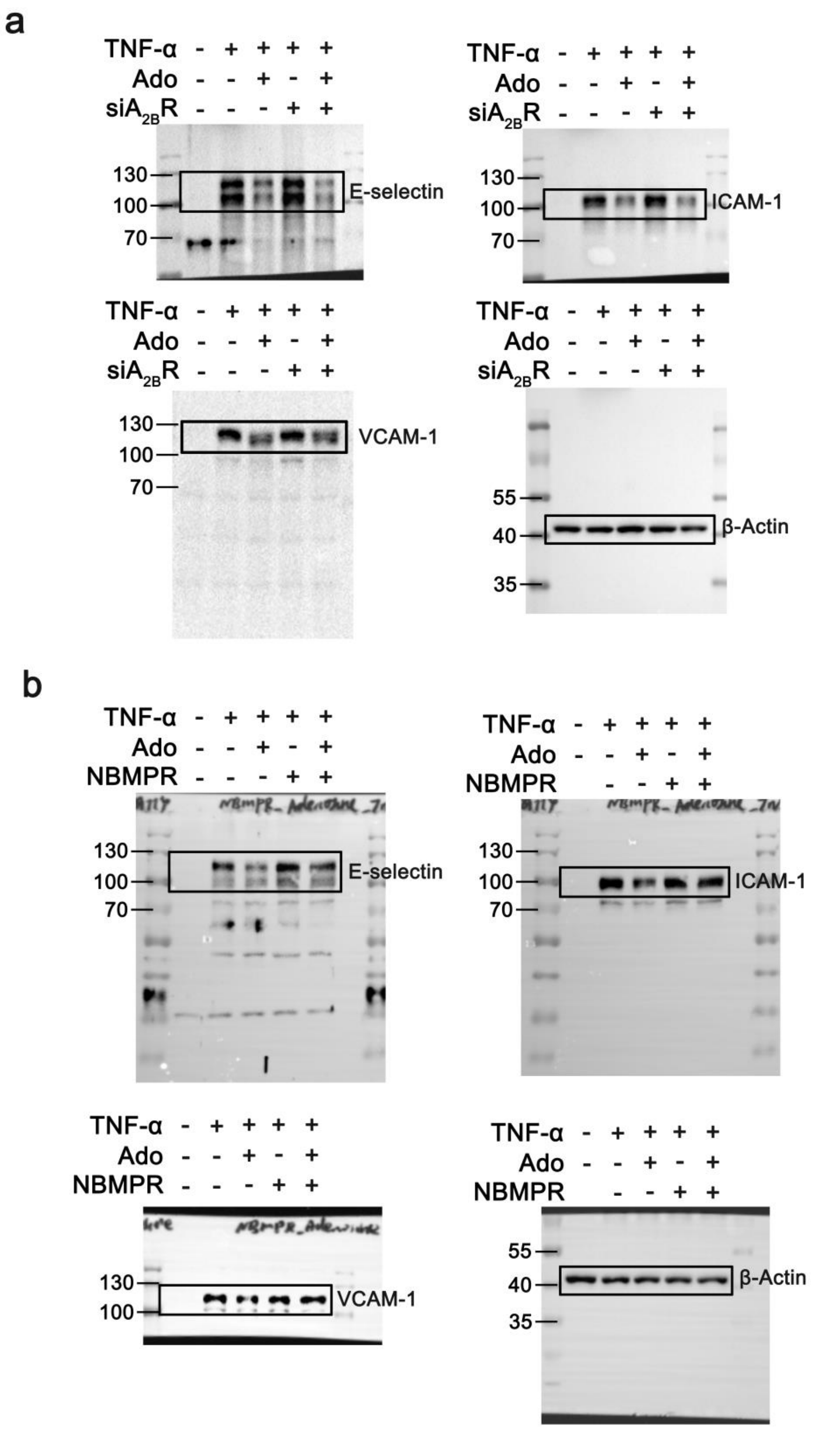

Supplementary Figure 13: Full gel scans for Fig. 2c and 2f. a, Gel scans for Fig. 2c. **b,** Gel scans for Fig. 2f.

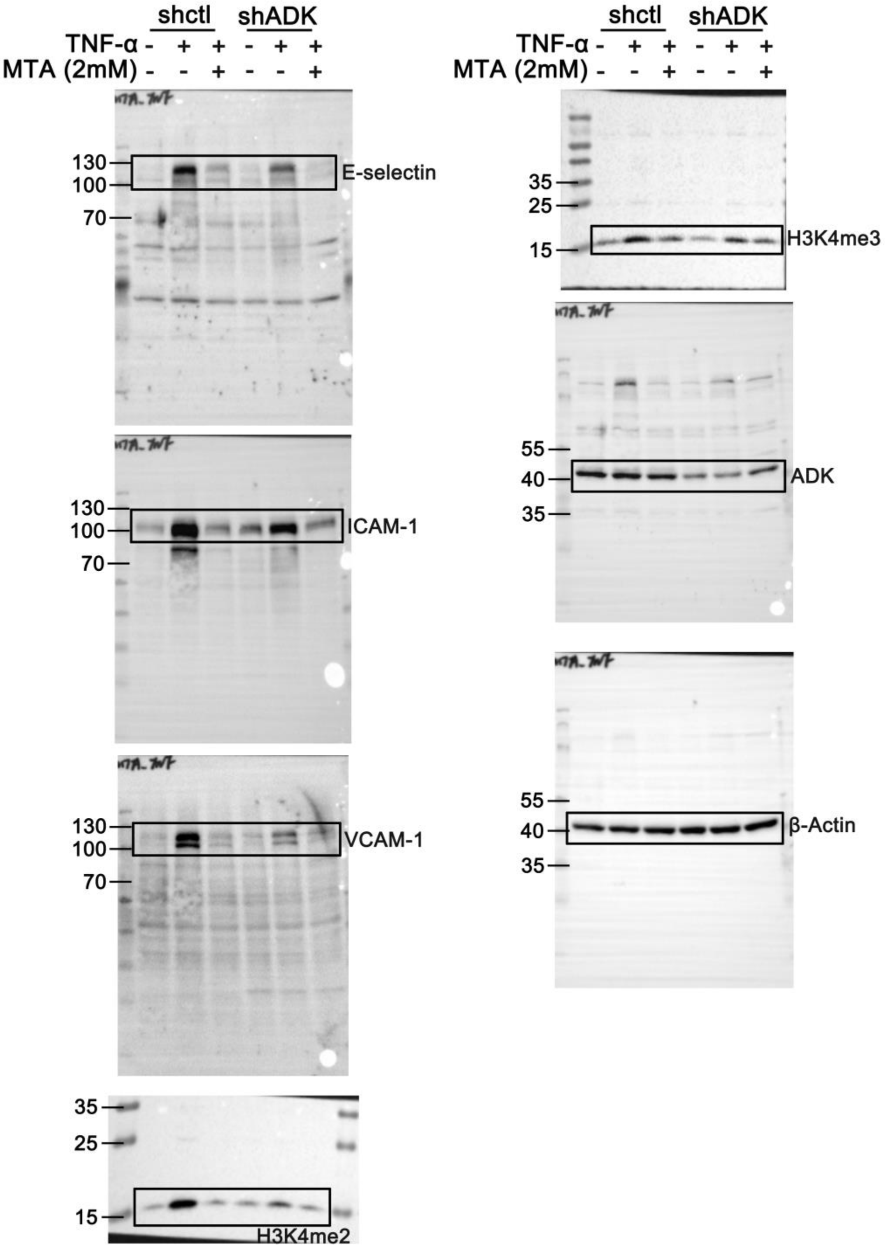

Supplementary Figure 14: Full gel scans for Fig. 4a.

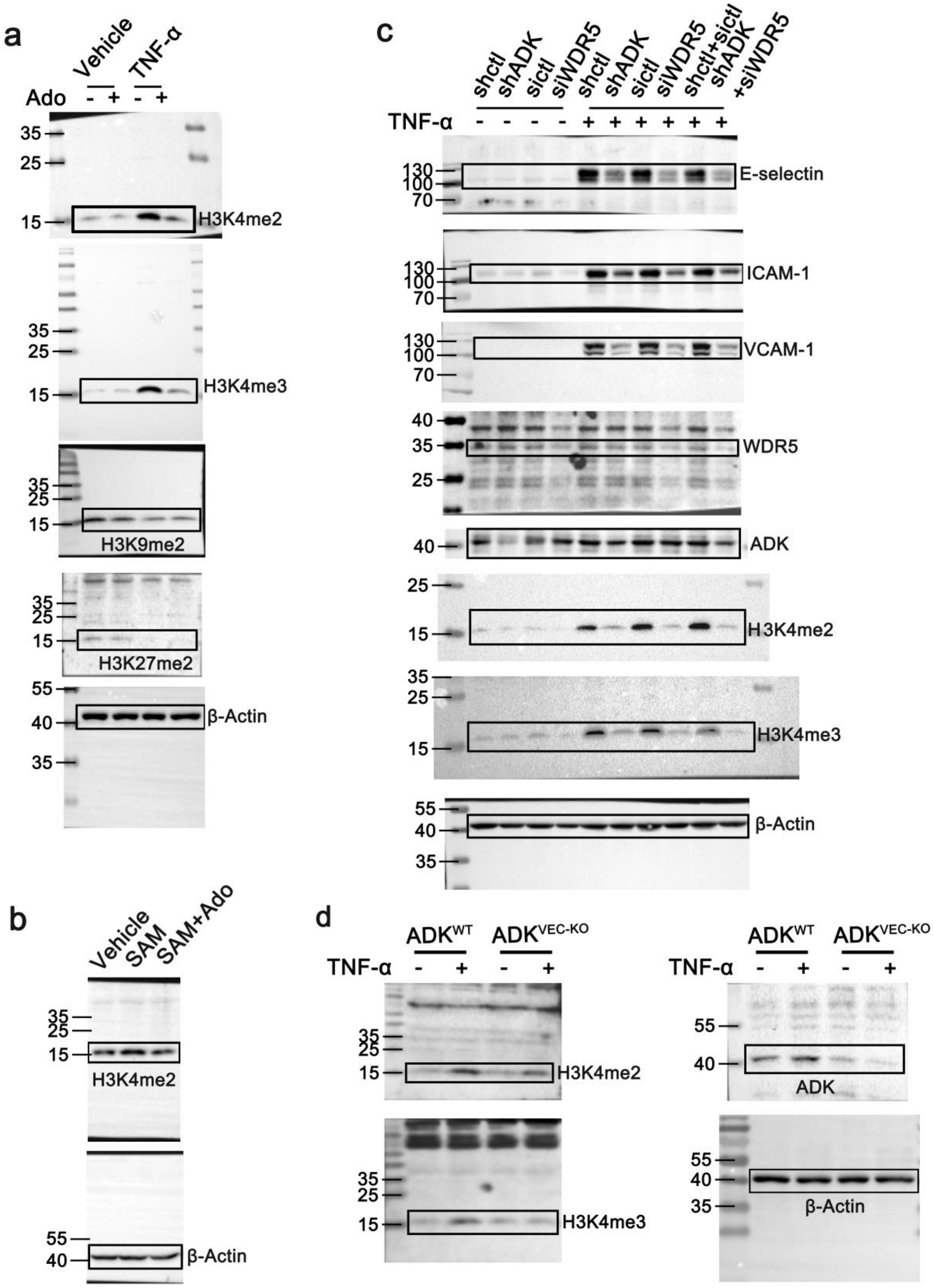

Supplementary Figure 15: Full gel scans for Fig. 4b, 4d, 4e and 4f. a, Gel scans for Fig. 4b. **b,** Gel scans for Fig. 4d. **c,** Gel scans for Fig. 4e. **d,** Gel scans for Fig. 4h.

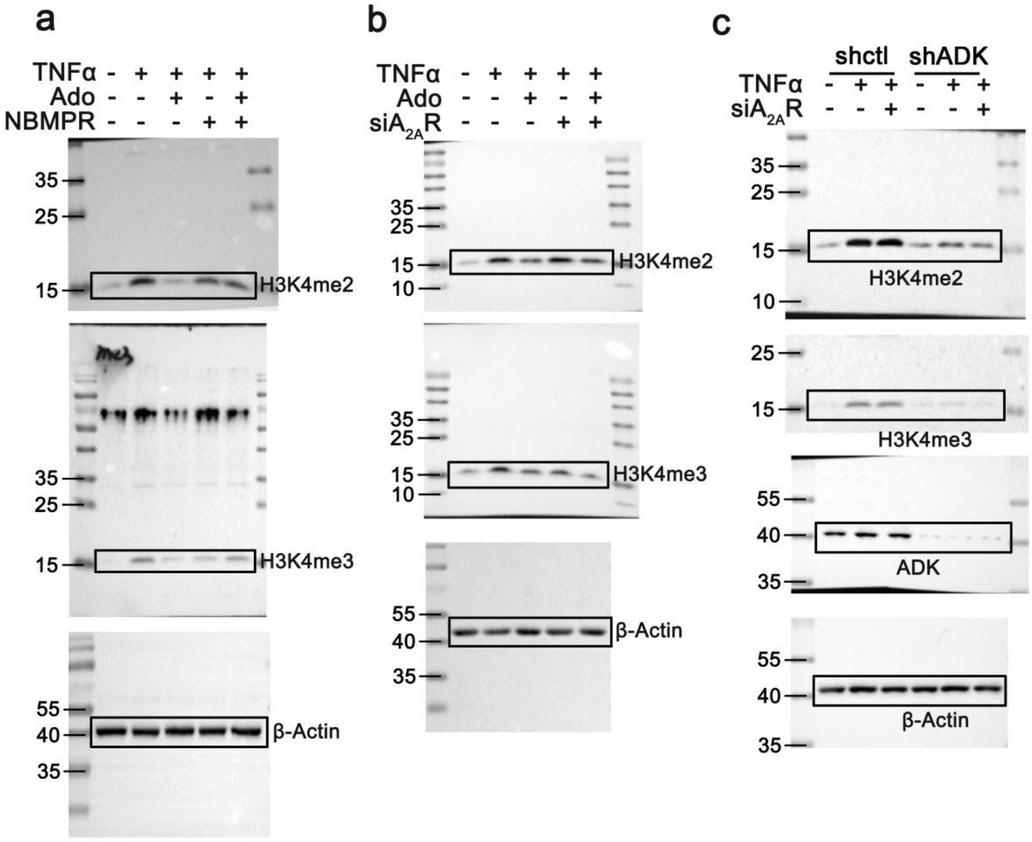

Supplementary Figure 16: Full gel scans for Fig. 4i, 4j and 4k. a, Gel scans for Fig. 4i. **b,** Gel scans for Fig. 4j. **c,** Gel scans for Fig. 4k.

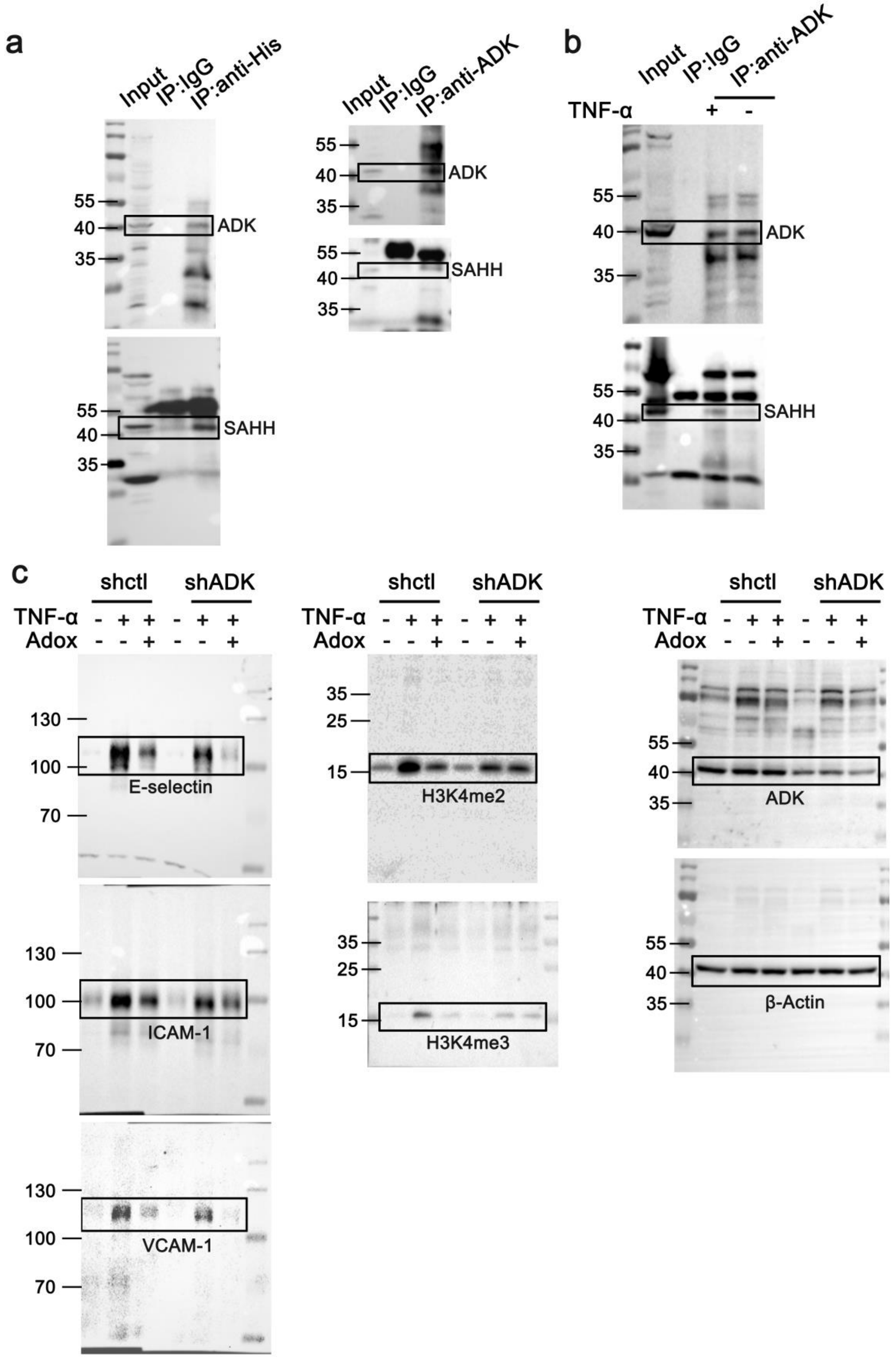

Supplementary Figure 17: Full gel scans for Fig. 5a, 5b and 5d. a, Gel scans for Fig. 5a. **b,** Gel scans for Fig. 5b. **c,** Gel scans for Fig. 5d.

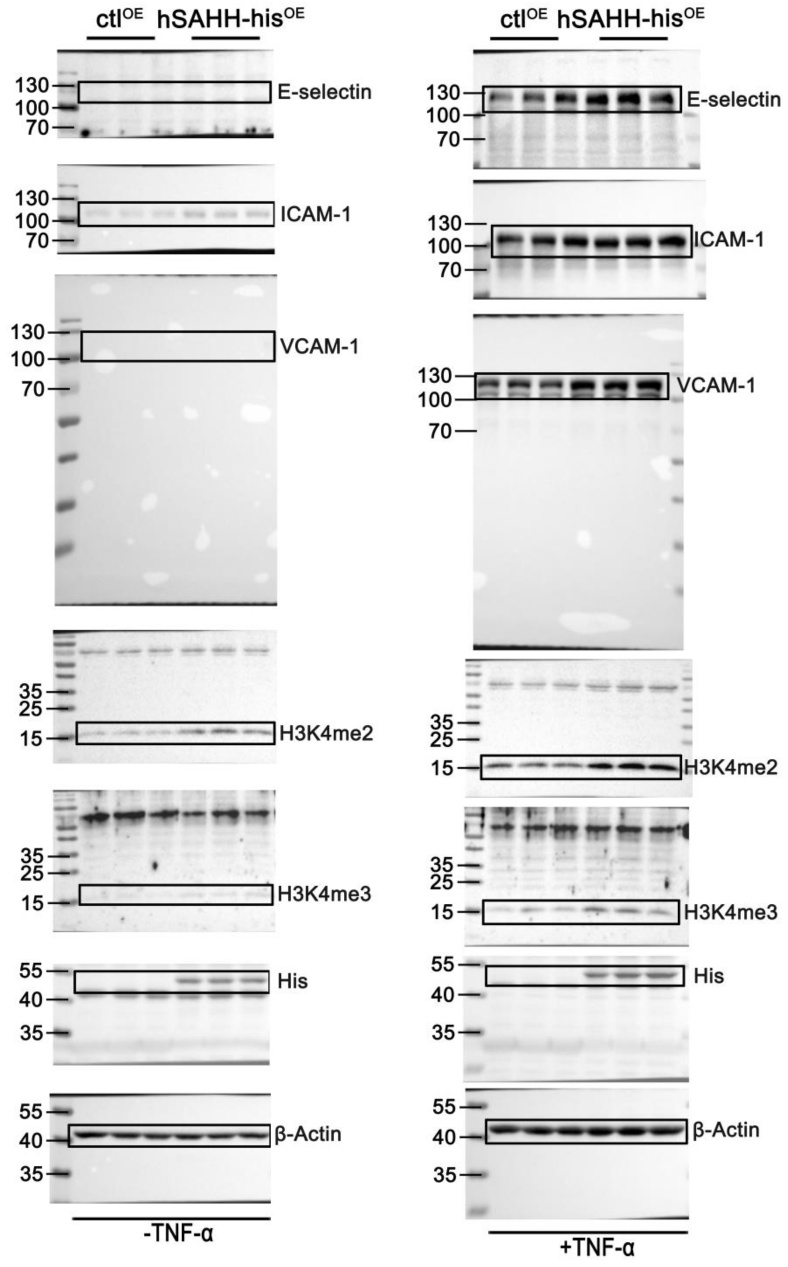

Supplementary Figure 18: Full gel scans for Fig. 5e.

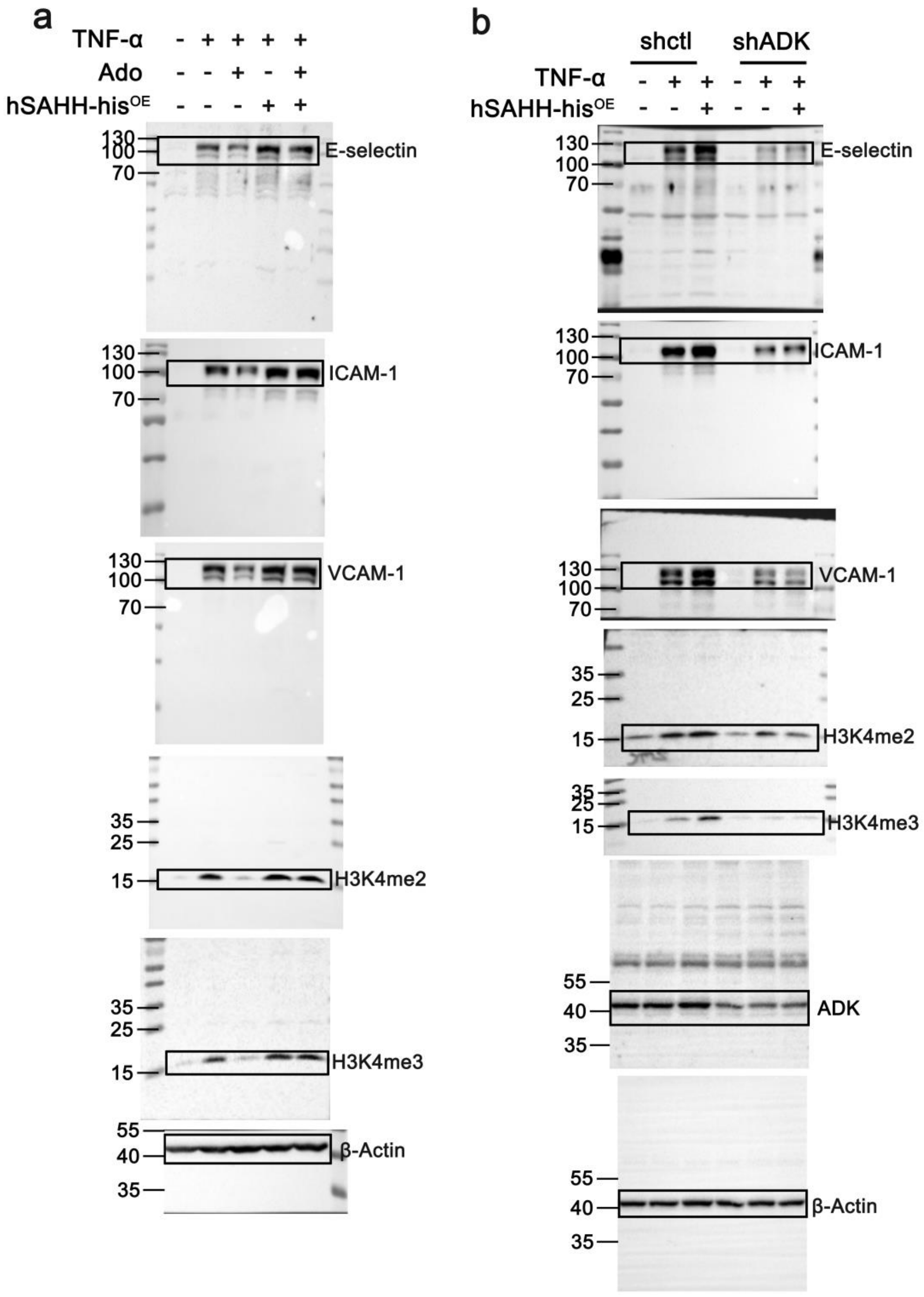

Supplementary Figure 19: Full gel scans for Fig. 5g and 5h. a, Gel scans for Fig. 5g. **b,** Gel scans for Fig. 5h.

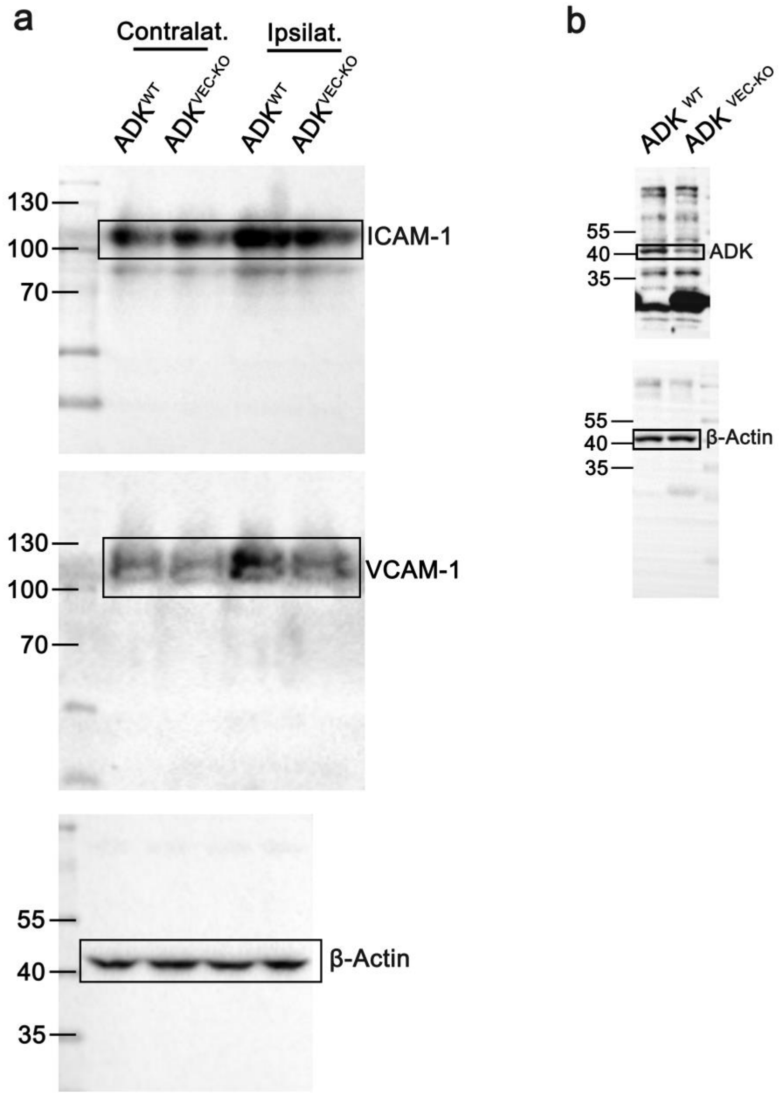

Supplementary Figure 20: Full gel scans for Fig. 7n and Supp. Fig 1c. a, Gel scans for Fig. 7n. **b,** Gel scans for Supp. Fig. 1c.

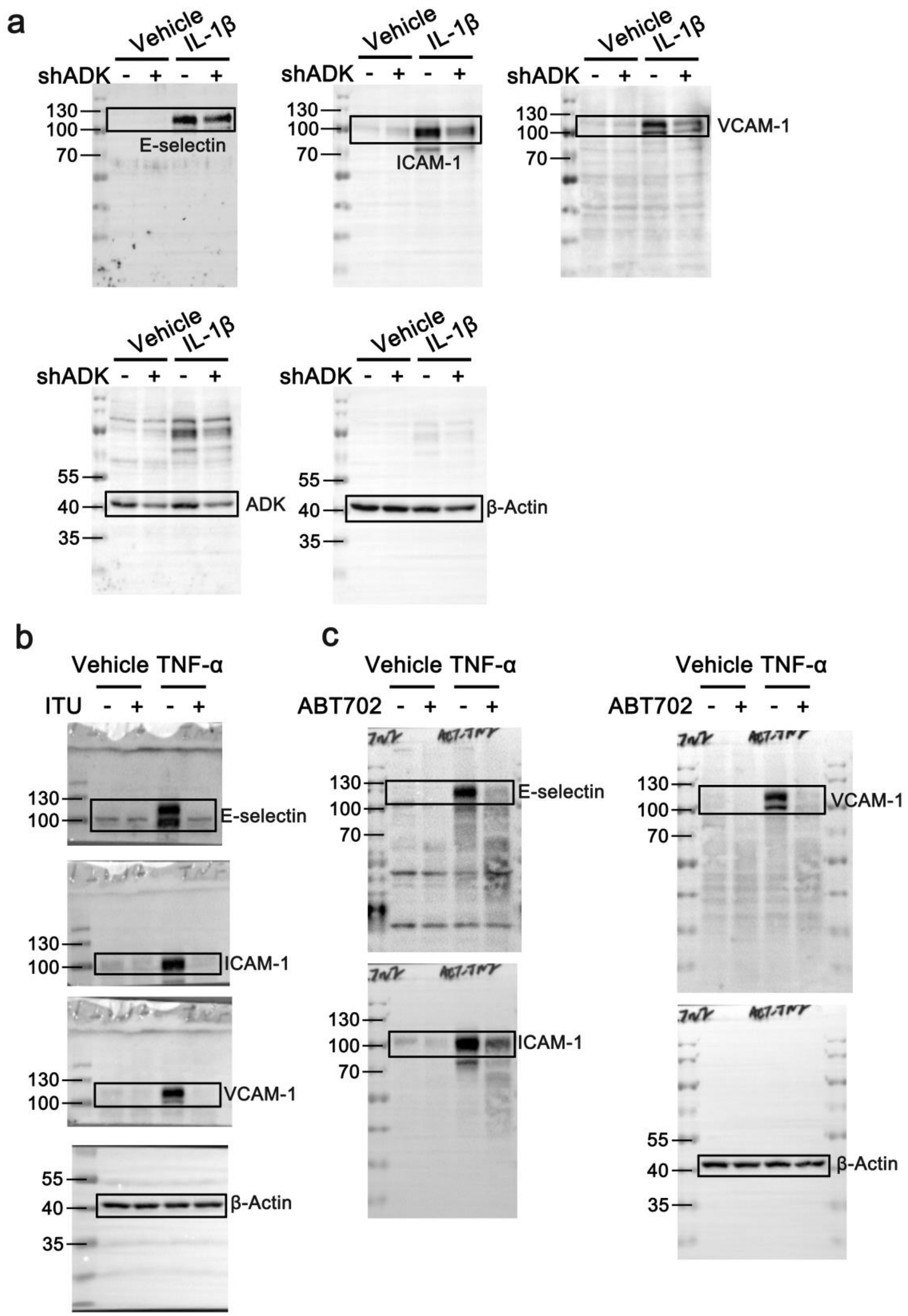

Supplementary Figure 21: Full gel scans for Supp Fig. 2e, 2f and 2g. a, Gel scans for Supp. Fig. 2e. **b,** Gel scans for Supp. Fig. 2f. **c,** Gel scans for Supp. Fig. 2g.

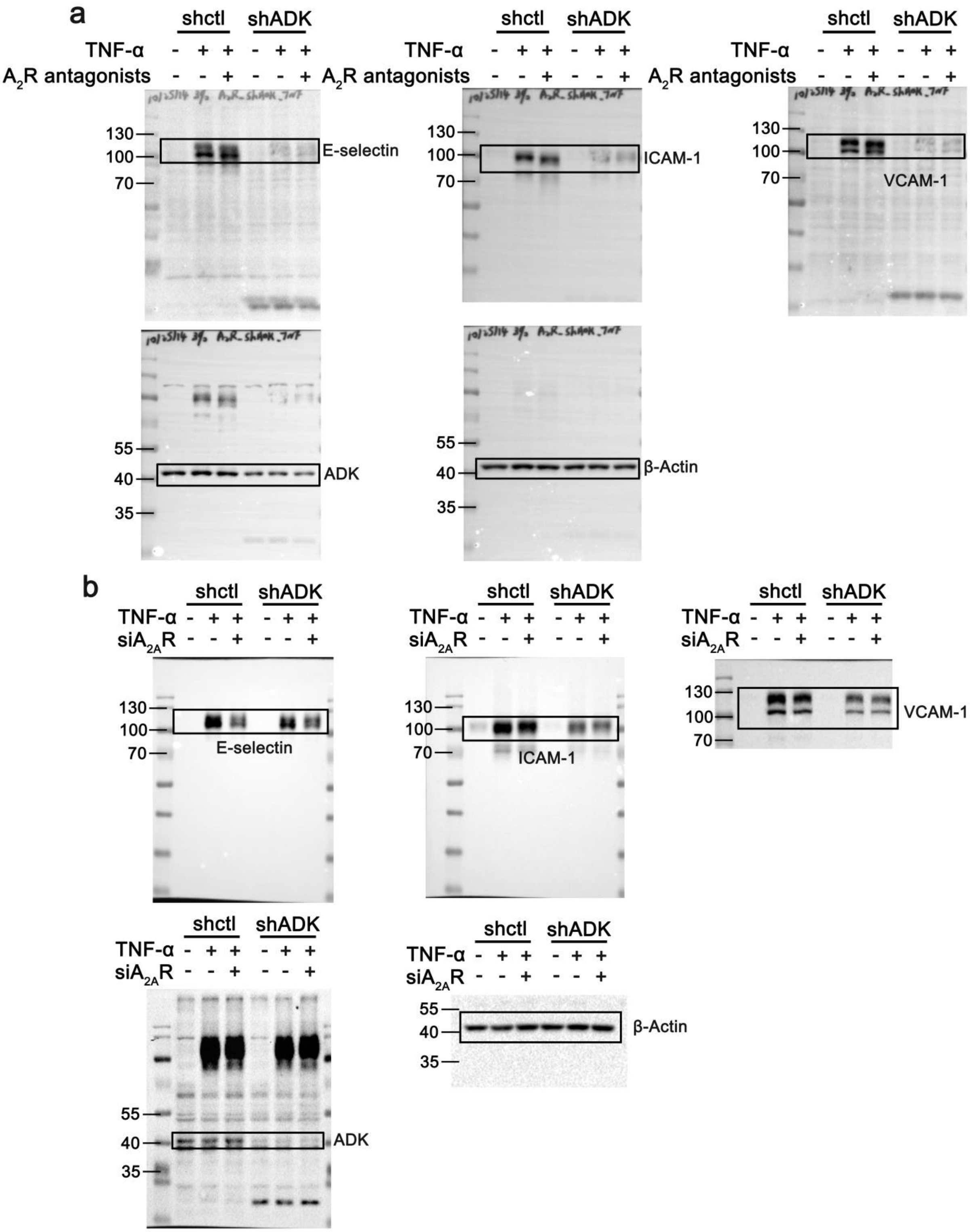

Supplementary Figure 22: Full gel scans for Supp Fig. 3a and 3b. a, Gel scans for Supp. Fig. 3a. **b,** Gel scans for Supp. Fig. 3b.

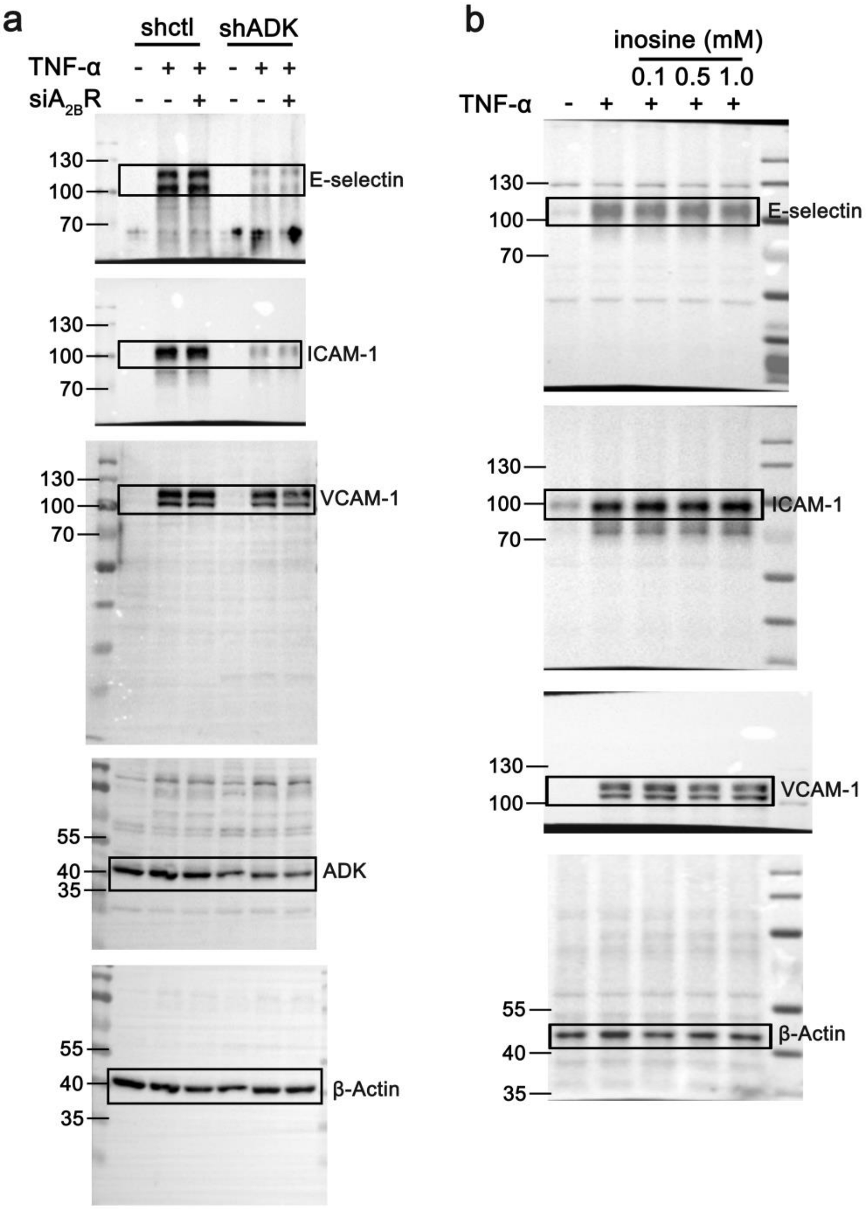

Supplementary Figure 23: Full gel scans for Supp Fig. 3c and 3d. a, Gel scans for Supp. Fig. 3c. **b,** Gel scans for Supp. Fig. 3d.

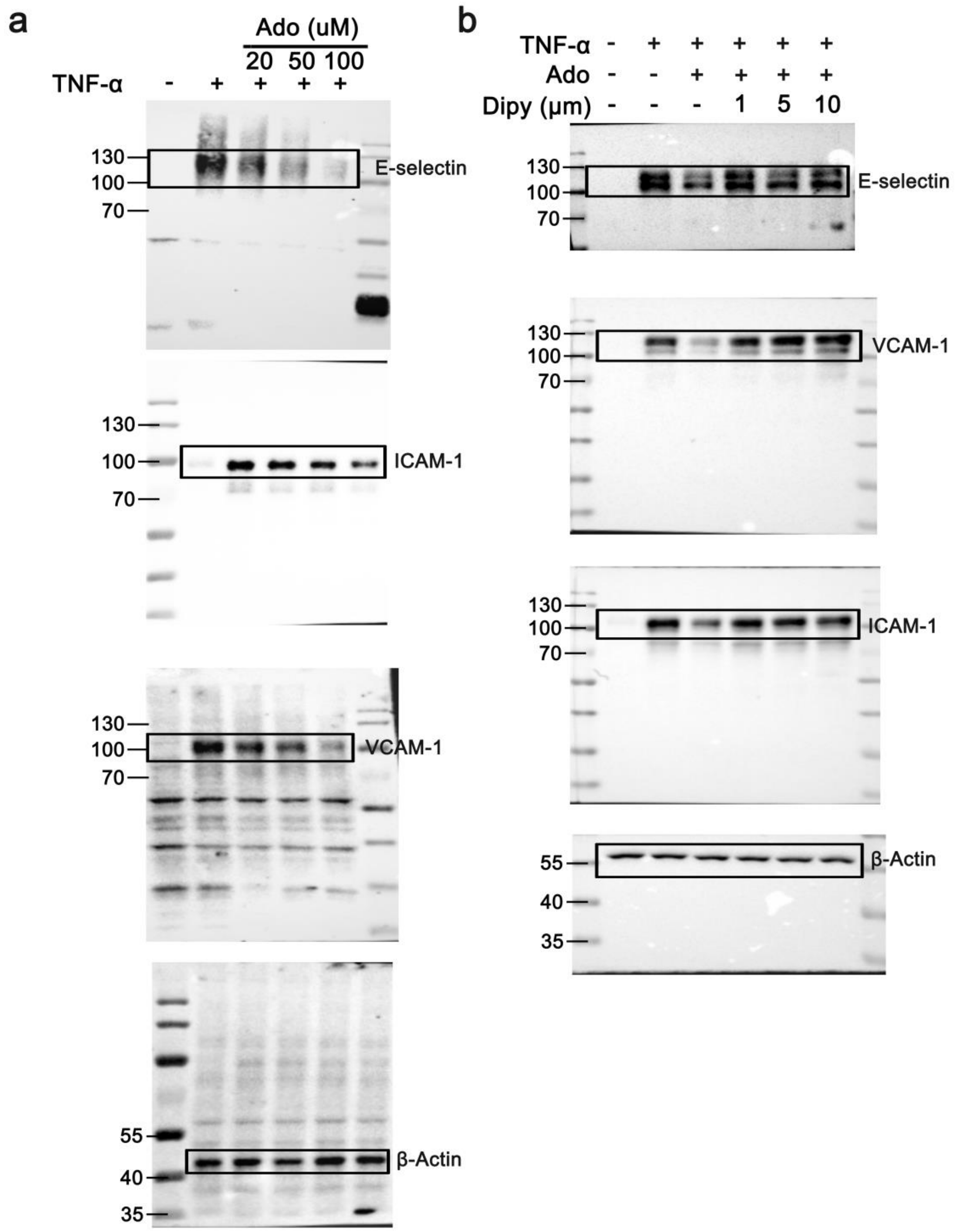

Supplementary Figure 24: Full gel scans for Supp Fig. 4a and 4c. a, Gel scans for Supp. Fig. 4a. **b,** Gel scans for Supp. Fig. 4c.

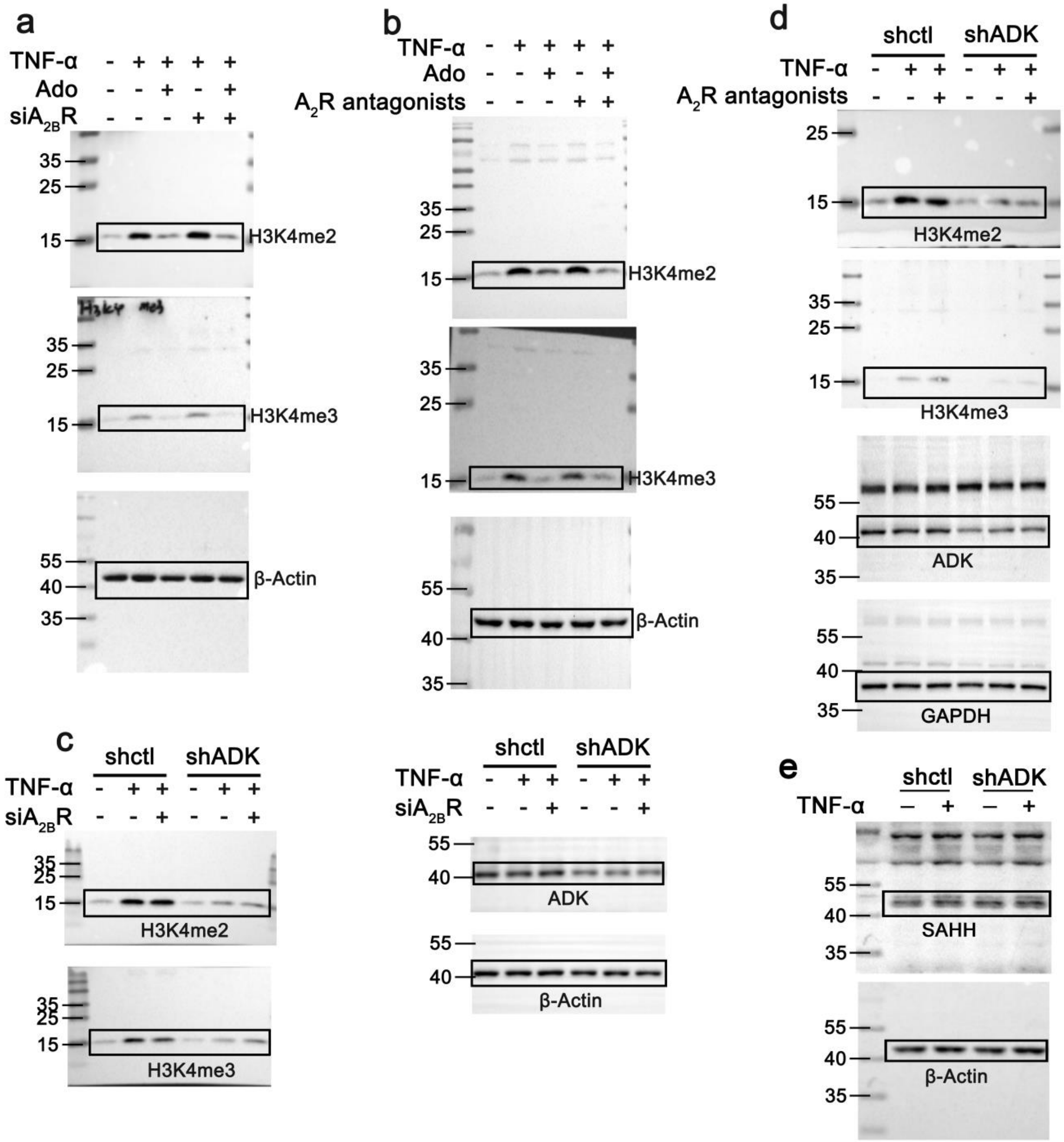

Supplementary Figure 25: Full gel scans for Supp Fig. 6a-6d. a, Gel scans for Supp. Fig. 6a. **b,** Gel scans for Supp. Fig. 6b. **c,** Gel scans for Supp. Fig. 6c. **d,** Gel scans for Supp. Fig. 6d. **e,** Gel scans for Supp. Fig. 6e.

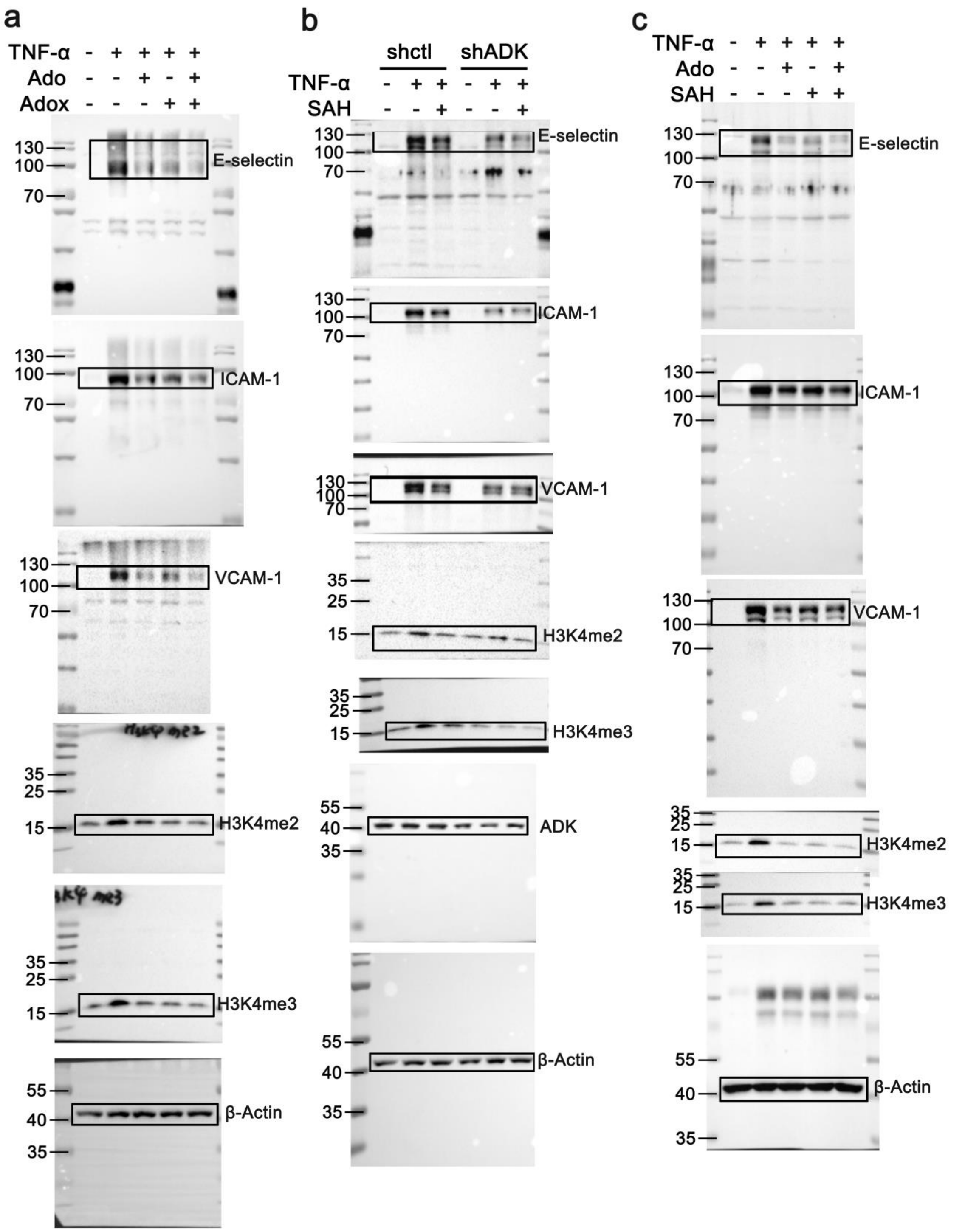

Supplementary Figure 26: Full gel scans for Supp Fig. 7a-7c. a, Gel scans for Supp. Fig. 7a. **b,** Gel scans for Supp. Fig. 7b. **c,** Gel scans for Supp. Fig. 7c.

Supplementary Table 1 Real-time PCR and ChIP real-time PCR primers

Gene name	Primer sequences
Human E-selectin	Forward: 5'- CCCGAAGGGTTTGGTGAG-3'
	Reverse: 5'- TAAAGCCCTCATTGCATTGA-3'
Human ICAM-1	Forward: 5'- GGCCGGCCAGCTTATACAC-3'
	Reverse: 5'- TAGACACTTGAGCTCGGGCA-3'
Human VCAM-1	Forward: 5'- TCAGATTGGAGACTCAGTCATGT-3'
	Reverse: 5'- ACTCCTCACCTTCCCGCTC-3'
Human IL-6	Forward: 5'- TCCTGCAGAAAAAGGCAAAG-3'
	Reverse: 5'- GCCCAGTGGACAGGTTTCT-3'
Human MCP-1	Forward: 5'- GTGAGGAACAAGCCAGAGCTG-3'
	Reverse: 5'- TGCGCAGAATGAGATGAGTTG-3'
Human IL-8	Forward: 5'- GACCACACTGCGCCAACAC-3'
	Reverse: 5'- CTTCTCCACAACCCTCTGCAC-3'
Human E-selectin Promoter (ChIP)	Forward: 5'-GGCCTCAGCCGAAGTAGTG-3'
	Reverse: 5'-CTGCTGCCTCTGTCTCAGG-3'
Human ICAM-1 Promoter (ChIP)	Forward: 5'-CCCTGCCACCGCCGCC-3'
	Reverse: 5'-AGGGGCGGTGCTGCTTTCC-3'
Human VCAM-1 Promoter (ChIP)	Forward: 5'-CCCATTTGCTCATTTGGTCTCAGC-3'
	Reverse: 5'-AAGGGTCTTGTTGCAGAGG-3'
